# Phenotypic and molecular characterization of novel pulmonary adenocarcinoma cell lines established from a dog

**DOI:** 10.1038/s41598-023-44062-1

**Published:** 2023-10-05

**Authors:** Kosuke Kobayashi, Reika Deja Takemura, Jiro Miyamae, Ikki Mitsui, Kohei Murakami, Kenji Kutara, Kohei Saeki, Teppei Kanda, Yasuhiko Okamura, Akihiko Sugiyama

**Affiliations:** Faculty of Veterinary Medicine, Okayama University of Science, Ikoino-oka 1-3, Imabari Ehime, Japan

**Keywords:** Cancer, Cell biology, Molecular biology, Stem cells, Oncology

## Abstract

Canine pulmonary adenocarcinoma (PAC) resembles human lung tumors in never-smokers, but it is rarer than human pulmonary adenocarcinoma. Therefore, research on canine PAC is challenging. In the present study, we successfully established various novel canine PAC cell lines from a single lesion in a dog, including two parent cell lines and fourteen cloned cell lines, and characterized their cellular properties in vitro. Several of these cell lines showed epithelial–mesenchymal transition (EMT)-like and/or cancer stem cell (CSCs)-like phenotypes. We additionally assessed the sensitivity of the cells to vinorelbine in vitro. Three clonal lines, two of which showed EMT- and CSC-like phenotypes, were resistant to vinorelbine. Furthermore, we evaluated the expression and activation status of EGFR, HER2, and Ras signaling factors. The findings indicated that the cell lines we established preserved the expression and activation of these factors to varying extents. These novel canine PAC cell lines can be utilized in future research for understanding the pathogenesis and development of treatments for canine PAC.

## Introduction

Canine primary lung tumors share clinicopathologic features with human lung tumors in never-smokers but is rare compared with that in humans^1^, with an incidence rate of 4.2–15 per 100,000 dogs per year^2,3^. Canine pulmonary adenocarcinoma (PAC) is the most frequently encountered tumor (87.1%), followed by sarcoma (7.6%), adenoma (3.2%), and pulmonary neuroendocrine tumor (1.5%) in dogs with primary pulmonary mass^4^. Locoregional tumors spread to other lung areas or lymph nodes, and distant metastasis via hematogenous and lymphatic routes are common in canine lung tumors. Metastasis is a crucial prognostic factor. In one study, the rates of local vascular or lymphatic invasion and distant metastases were 71% and 23%, respectively^5^. Surgical resection is the first choice of treatment, and patients without metastasis appear to have a favorable prognosis. However, the prognosis for advanced disease is very poor, and the median survival time has been reported to be less than 1 year^4,6^. In humans, cisplatin-based chemotherapy combined with surgery is available and effective in patients with non-small-cell lung cancer^7^. However, the efficacy of chemotherapy in dogs with lung tumors remains unclear. In a previous study, two of seven canine PAC cases partially responded to vinorelbine, a vinca alkaloid with high transferability to the lung tissue^8^. Toceranib, a multi-target tyrosine kinase inhibitor, has been reported to be partially effective in some canine PAC cases^9,10^. However, reliable clinical trials with larger sample sizes are lacking because cases of naturally developed PAC are rare. Moreover, only a few types of canine PAC cell lines are currently available^11–13^, and their cellular characteristics have not been clearly described. Therefore, a novel experimental model is required for further research on canine PAC.

Epithelial–mesenchymal transition (EMT) is a key event in tumor progression and metastasis. During EMT, tumor cells acquire mesenchymal phenotypes, including fibroblast-like morphology, high motility, and vimentin expression, resulting from the downregulation of epithelial hallmarks, including adhesion properties and E-cadherin expression^14,15^. Once EMT occurs, tumor cells migrate from the primary lesion to release circulating tumor cells into the bloodstream. Subsequently, mesenchymal–epithelial transition occurs, followed by metastasis. In human breast and pancreatic cancer models, EMT has been reported to be indispensable for lung metastasis, and it contributes to chemoresistance^16,17^. Other reports have also described the contribution of EMT to chemoresistance in human oncology^18^. Although the role of EMT in metastasis is still under discussion, the importance of EMT in cancer therapy in dogs has been described in a previous review^19^.

The relationship between EMT and cancer stem cells (CSCs), which are tumor-initiating cells, has been investigated^18,20^. CSCs are small subsets with extensive self-renewing properties that drive tumorigenesis in tumor tissues, and they are crucial factors in tumor metastasis, recurrence, and chemoresistance^21,22^. CSCs have mesenchymal properties and invasive features, the hallmarks of EMT. CSCs can be detected using various techniques, including surface antigen analysis such as CD133 analysis, colony- or sphere-formation assays, aldehyde assays, and side population (SP) assays with Hoechst 33342^22,23^ or Vybrant DyeCycle Violet (DCV)^24^. CSCs have been detected in various types of human tumors^22–24^. In canine tumors, CSCs have been detected in mammary carcinoma^26^, osteosarcoma^27^, and PAC^11^.

In humans, personalized treatment for PAC is based on the molecular subtypes of oncogene mutations and activation of tumor-associated proteins^28^. The epidermal growth factor receptor (EGFR) and human epidermal growth factor receptor 2 (HER2), the preferred heterodimer partner of EGFR, are cell surface receptor tyrosine kinases (RTK), and their activation promotes tumor cell proliferation. Their activating mutation and overexpression have been observed in various types of tumors, including human lung cancer^29,30^. In canine PAC, the activation of EGFR and HER2 has been reported in tumor tissue^31–33^. The small G protein, Ras, is an oncogenic factor. Its activating mutation has been detected in various types of tumors, including human and canine PAC^34,35^. Prenylated Ras translocates from the cytoplasm to the membrane, and it is activated by RTK, such as EGFR. Subsequently, activated Ras stimulates the downstream mitogen-activated protein kinase (MAPK) and phosphoinositide-3 kinase (PI3K) pathways, which promote cell proliferation. These factors are recognized as potent therapeutic targets in malignant tumors, including PAC^36^; however, they have not been sufficiently investigated in canine PAC.

In the present study, we established two types of canine PAC cell lines under different culture conditions from a dog with naturally occurring PAC. Fourteen cloned cell lines with various morphological characteristics were established by single-cell cloning. We assessed their proliferative capacities in vitro and their characteristics, focusing on the EMT and CSC phenotypes. Additionally, sensitivity to vinorelbine in vitro was assessed. Finally, we evaluated the expression of several tumor-associated proteins.

## Results

### Establishment of cell lines

We initiated two primary cultures from canine PAC tissue under different culture conditions, and named them CPACd-p and CPACr-p cell lines. They were cultured in complete media based on Dulbecco’s modified Eagle’s medium (DMEM) and Roswell Park Memorial Institute (RPMI) 1640 medium, respectively, and were successfully purified and propagated for more than 50 passages.

Next, we cloned CPACd-p and CPACr-p using a limiting-dilution method. Fifty-three and thirty-four clones were successfully obtained from the CPACd-p and CPACr-p cells, respectively. Seven clones were selected from each of these groups according to their morphology and subculture intervals. The other clones which were not selected had similar characters to the selected clones. Only the 14 clones that were realistic in terms of number of analyses were used in subsequent experiments. The selected cloned cells were propagated for more than 30 passages (CPACd-AB4, CPACd-AC12, CPACd-AD4, CPACd-AE3, CPACd-BB4, CPACd-CG11, and CPACd-DC10; CPACr-AD4, CPACr-AH4, CPACr-BC9, CPACr-BH12, CPACr-CB2, CPACr-DC3, and CPACr-DD10).

### Microscopic morphology

**Figure 1:**
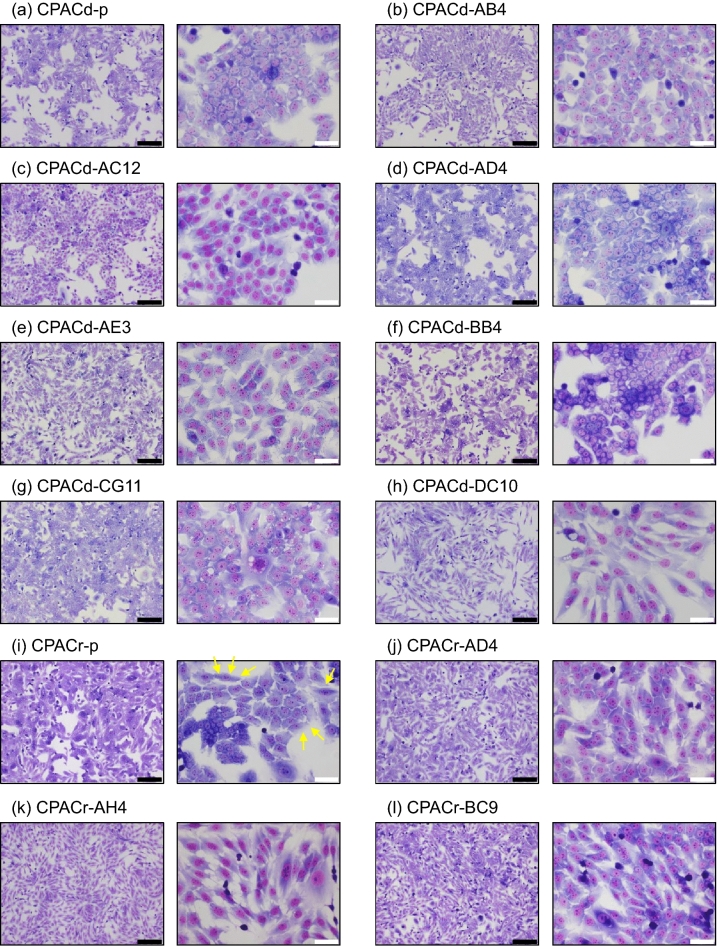

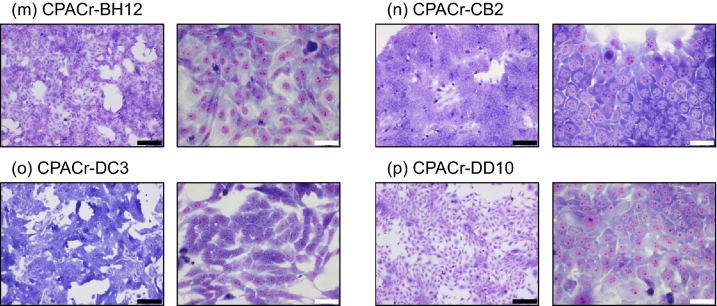
Morphology of the canine PAC cell lines. Cells were stained with Wright–Giemsa stain. Left and right panels in every figure (**a**–**p**) are images captured under a light microscope at 40 × and 400 × magnification, respectively. Scale bar: black, 500 µm; white, 50 µm. Yellow arrows in (i) indicate mesenchymal-like cells.

Images obtained under a light microscope are shown in Fig. 1 and Supplementary Fig. S1. The CPACd-p cell line showed epithelial cellular morphology with a small, round shape. In contrast, the CPACr-p cell line contained cells with different shapes, including cells with epithelial and mesenchymal-like morphologies. In the cloned cell lines, CPACd-DC10 and CPACr-AH4 cells showed a mesenchymal-like morphology with a spindle shape. CPACd-AE3, CPACr-BC9, CPACr-BH12, and CPACr-DC3 cells showed intermediate morphologies between the epithelial and mesenchymal morphologies. The other cell lines showed epithelial morphologies; however, the size of the cells and the adhesive properties, including the gap size between cells, were different between the cell lines.

### Growth of the cells in vitro

Additionally, we measured the doubling time of each cell line (Table 1, Supplementary Fig. S2). No significant differences were found between the CPACd-p and CPACr-p cells, of which doubling times were 23.3 ± 1.0 and 23.0 ± 0.1 h, respectively. CPACd-AB4 showed a substantially lower proliferative capacity than the parent cell line CPACd-p. In contrast, CPACd-BB4, CPACr-AD4, CPACr-BC9, and CPACr-DC3 exhibited substantially higher proliferative capacities than their parent cell lines (Dunnett’s test).

**Table 1:** Doubling times of the canine PAC cell lines.

		Doubling time (h)
CPACd	p	23.3 ± 1.0
	AB4	35.2 ± 1.4
	AC12	22.4 ± 1.2
	AD4	21.8 ± 2.0
	AE3	23.3 ± 2.0
	BB4	19.1 ± 1.1
	CG11	22.0 ± 1.2
	DC10	20.4 ± 1.1
CPACr	p	23.0 ± 0.1
	AD4	18.9 ± 0.9
	AH4	20.5 ± 2.2
	BC9	17.8 ± 0.7
	BH12	24.1 ± 1.8
	CB2	20.7 ± 1.0
	DC3	18.4 ± 2.3
	DD10	23.9 ± 0.9

### EMT phenotype

**Figure 2:**
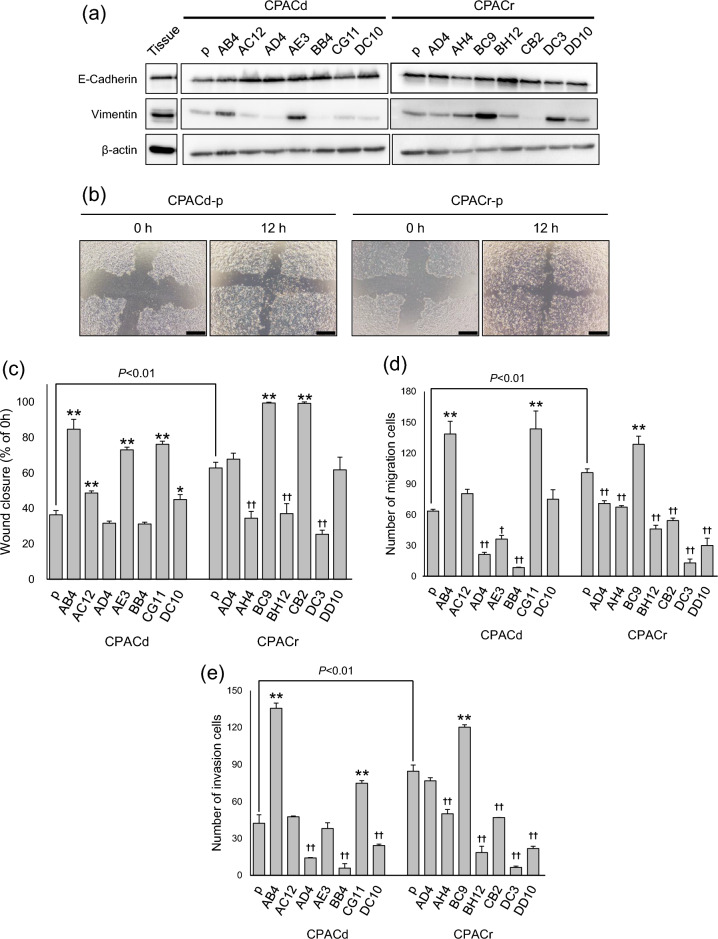
EMT phenotypes in the canine PAC cell lines. (**a**) The epithelial marker (E-cadherin) and mesenchymal marker (Vimentin) were assessed by immunoblotting. Full image of immunoblotting is shown in Supplementary information. Cell migration abilities were assessed by (**b**, **c**) wound-healing and (**d**) trans-membrane assays. Representative images of wound-healing assay in the parent cell lines are shown in (**b**). Scale bar: 500 µm. (**e**) Cell-invasion abilities were assessed by Matrigel invasion assay. Data are represented as the mean ± SD. Results are representative of three independent experiments. *P < 0.05, **P < 0.01: The migration or invasion ability is significantly higher than that of the parent cell lines; † *P* < 0.05, †† *P* < 0.01: The migration or invasion ability is significantly lower than that of the parent cell lines.

Since some cell lines showed a mesenchymal-like morphology, we hypothesized that EMT occurred in these cell lines. First, we assessed the expression of the epithelial and mesenchymal markers, E-cadherin and vimentin, respectively, in the tumor tissue and in each cell line (Fig. 2A). E-cadherin expression was observed in all samples, and no loss of expression was observed in any cell line. CPACr-p showed slightly higher expression of vimentin than CPACd-p. In the cloned cell lines CPACd-AB4, CPACd-AE3, CPACr-AH4, CPACr-BC9, and CPACr-DC3, vimentin expression was relatively high. Next, we assessed cellular motility in vitro, a phenotype of EMT. In the wound-healing assay, the migration ability of CPACr-p cells was significantly higher than that of CPACd-p cells (Student's t test, *P* = 0.00074; Fig. 2B,C, Supplementary Fig. S3). Among the cloned cell lines, CPACd-AB4, CPACd-AC12, CPACd-AE3, CPACd-CG11, CPACd-DC10, CPACr-BC9, and CPACr-CB2 cells showed substantially higher migration abilities than the parent cell lines. In contrast, the migration abilities of CPACr-AH4, CPACr-BH12, and CPACr-DC3 were substantially lower than that of the parent cell lines. In the Matrigel invasion assay, the migration and invasion abilities of CPACr-p cells were greater than that of CPACd-p cells (Student’s t test, *P* = 0.0022; Fig. 2D,E, Supplementary Figs. S4 and S5). Among the cloned cell lines, CPACd-AB4, CPACd-CG11, and CPACr-BC9 cells showed substantially higher migration and invasion abilities than the parent cells. In contrast, in CPACd-AD4, CPACd-AE3, CPACd-BB4, and all the cloned cell lines from CPACr-p, except for CPACr-BC9, the migration abilities were substantially lower than that of the parent cell lines. In CPACd-AD4, CPACd-BB4, CPACd-DC10, and all the cloned cell lines from CPACr-p, except for CPACr-AD4 and CPACr-BC9, the invasion abilities were substantially lower than that of the parent cell lines. In summary, the occurrence of EMT was highly suspected in CPACd-AB4 and CPACr-BC9, and possibly in CPACd-CG11.

### CSC phenotype

**Figure 3:**
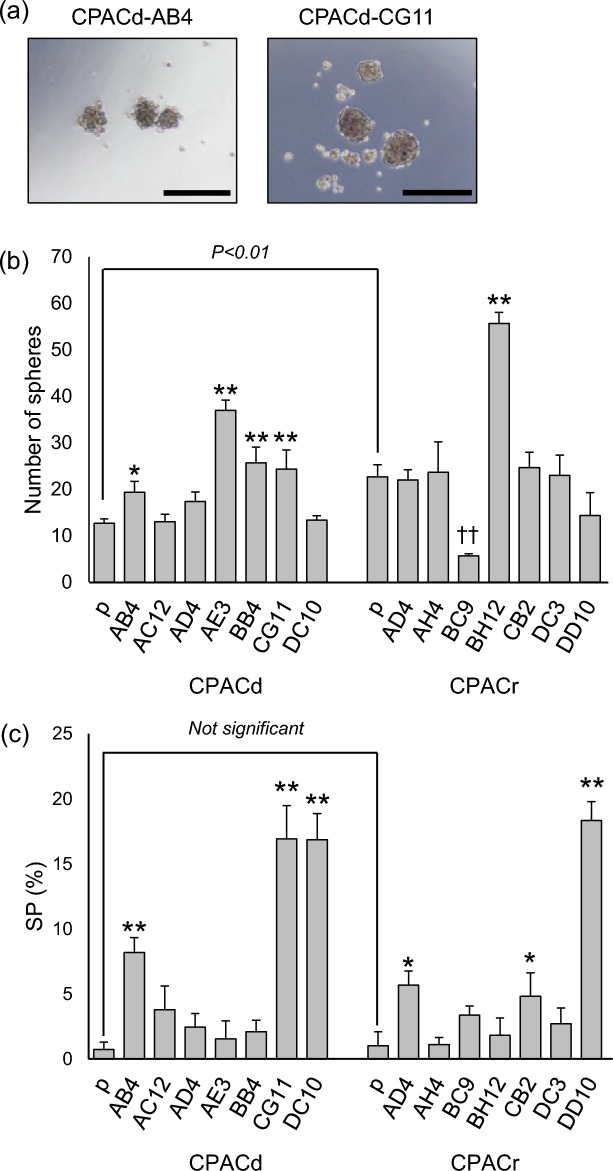
CSCs phenotypes in the canine PAC cell lines. (**a**, **b**) Sphere-formation abilities were assessed in low-attachment culture conditions. Cells were seeded at 2000 cells/well, and the number of spheres were counted visually under a light microscope at 40 × magnification after 10 days. Representative images of spheres in CPACd-AB4 and CPACd-CG11 are shown in (**a**). Scale bar: 500 µm. Data are represented as the mean ± SD. Results are representative of three independent experiments. (**c**) SP populations in each cell lines were assessed using Vybrant DyeCycle Violet (DCV). Cells (5 × 10^5^) were labeled with 5 µM DCV alone or in combination with 50 µM verapamil. SP populations were detected using flow cytometer with violet filter (450/40 and 660/20). Results are represented as the mean ± SD of three independent experiments. *P < 0.05, **P < 0.01: The number of spheres and SP populations are significantly higher than that of the parent cell lines; †† *P* < 0.01: The number of spheres is significantly lower than that of the parent cell lines.

To assess the CSC phenotype, we first evaluated the sphere-forming abilities of the cell lines (Fig. 3A,B). CPACr-p exhibited a significantly higher sphere-forming ability than CPACd-p (Student’s t test, *P* = 0.0071; Fig. 3B and Supplementary Fig. S6). The CPACd-AB4, CPACd-AE3, BB4, CPACd-CG11, and CPACr-BH12 cells showed substantially higher sphere-forming abilities than the parent cells. In contrast, the sphere-forming ability of the CPACr-BC9 cells was substantially lower than that of the parent cells. Next, using the DNA-binding dye DCV, we assessed the population of SP cells, which were recognized as CSCs, in the cell lines. SP cells were present at very low levels in the parent cell lines, CPACd-p and CPACr-p (Fig. 3C and Supplementary Fig. S7). In contrast, the cloned cell lines CPACd-AB4, CPACd-CG11, CPACd-DC10, CPACr-AD4, CPACr-CB2, and CPACr-DD10 contained substantially more SP cells than the parent cells. In summary, high levels of CSCs were possibly present in the CPACd-AB4, CPACd-CG11, and CPACr-AD4 cells.

### Resistance to vinorelbine in vitro

**Figure 4:**
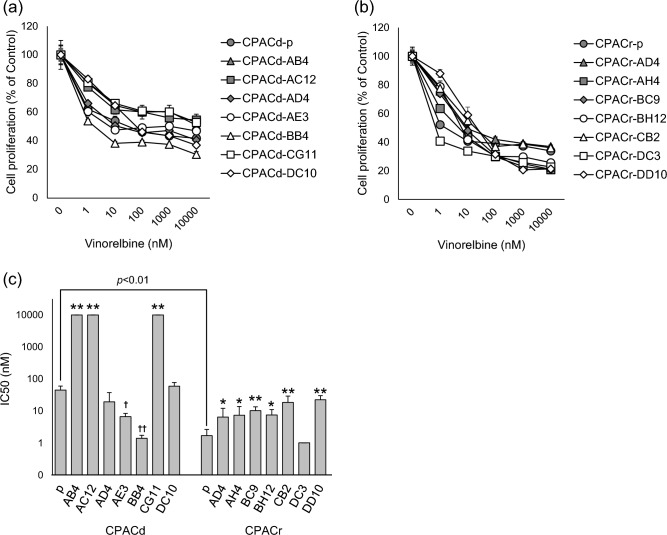
Sensitivity to vinorelbine in the canine PAC cell lines. (**a**) CPACd cell lines and (**b**) CPACr cell lines were treated with vinorelbine (0–10,000 nM) for 48 h. Cell proliferation was measured using cell counting kit-8. Data are represented as the percentage of control (0 nM; mean ± SD). Results are representative of three independent experiments. (**c**) The IC50 are calculated in each cell line. Results are represented as the mean ± SD of three independent experiments. *P < 0.05, **P < 0.01: The IC50 is significantly higher than the parent cell lines; † *P* < 0.05, †† *P* < 0.01: The IC50 is significantly lower than that of the parent cell lines.

We assessed sensitivity to vinorelbine, a semisynthetic vinca alkaloid commonly used in human patients with lung tumors, in the cell lines in vitro. Vinorelbine exerted anti-proliferative effects in a concentration-dependent manner in all cell lines (Fig. 4A,B). The IC50 values summarized in Supplementary Table S1. CPACr-p was significantly more sensitive to vinorelbine than was CPACd-p (Student's t test, *P* = 0.0099). CPACd-AB4, CPACd-AC12, CG11, and all cloned cell lines from CPACr-p, except for CPACr-DC3, were less sensitive to vinorelbine than their parent cell lines. In contrast, CPACd-AE3 and CPACd-BB4 were more sensitive than the parent cell line CPACd-p. The IC50 values of vinorelbine in CPACd-AB4, CPACd-AC12, and CPACd-CG11 were higher than 10 µM, suggesting that these cell lines were resistant to vinorelbine treatment. In contrast, CPACd-BB4, CPACr-p, and CPACr-DC3 were relatively sensitive (Fig. 4C).

### Expression of tumor-associated proteins

**Figure 5:**
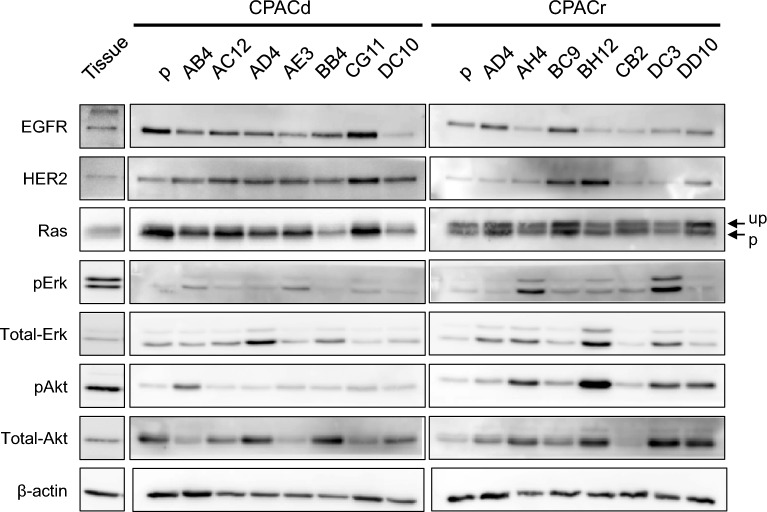
Expression profiles of malignancy-associated proteins in canine PAC cell lines. Expression and phosphorylation status of indicated proteins were assessed by immunoblotting. Results are representative of three independent experiments. Full image of immunoblotting is shown in Supplementary information.

We evaluated the expression and phosphorylation status of several tumor-associated proteins in the cell lines using immunoblotting (Fig. 5). EGFR and HER2 were expressed in all cell lines. CPACd-CG11 showed relatively high expression of both EGFR and HER2. High Ras prenylation was observed, particularly in the CPACd cell lines, compared to that in CPACr cell lines. High phosphorylation levels of Erk1/2 were observed in CPACr-AH4 and CPACr-DC3. Akt phosphorylation status was relatively high in CPACd-AB4, CPACr-AH4, CPACr-BH12, CPACr-DC3, and CPACr-DD10. In tumor tissues, EGFR and HER2 expression levels were lower than that in the cell lines. Additionally, Ras prenylation and phosphorylation of the downstream factor Erk1/2 were low. Additionally, the levels of Akt phosphorylation were high.

## Discussion

In the present study, we successfully established various canine PAC cell lines from a dog. Cellular morphology varies among cell lines, including epithelial, mesenchymal, and intermediate types. Moreover, certain cell lines were suspected to have developed EMT with increased vimentin expression and high motility, and they showed CSC-like phenotypes characterized by high sphere-forming ability and high levels of SP cells. Sensitivity to vinorelbine in vitro varied among the cell lines. These findings are summarized in Table 2. CPACd-AB4 and CPACd-CG11 exhibited both EMT- and CSC-like phenotypes and showed resistance to vinorelbine. Therefore, the two clones may have high malignancy rates among the newly developed cell lines in the present study.

**Table 2:** Characteristics of the canine PAC cell lines.

	CPACd								CPACr							
	p	AB4	AC12	AD4	AE3	BB4	CG11	DC10	p	AD4	AH4	BC9	BH12	CB2	DC3	DD10
Cellular morphology^a^	E	E	E	E	I	E	E	M	E, I	E	M	I	I	E	I	E
Proliferative capasity^b^	++	+	++	++	++	+++	++	++	++	+++	++	+++	++	++	+++	++
Expression of vimentin	+	++	+	−	++	−	+	+	+	+	+	++	+	−	++	+
EMT																
Migration ability																
(WHA)^c^	+	++	+	±	++	±	++	+	+	+	±	++	+	++	++	+
(TMA) ^d^	+	++	+	±	±	±	++	+	+	+	+	++	±	±	±	±
Invasion ability^d^	±	++	±	±	±	±	+	±	+	+	±	++	±	±	±	±
CSC																
Sphere forming ability ^e^	±	+	±	+	++	+	+	±	+	+	+	±	++	+	+	±
Population of SP^f^	±	+	±	±	±	±	++	++	±	+	±	±	±	±	±	++
Sensitivity to vinorelbine^g^	+	±	±	+	++	++	±	+	++	++	++	+	++	+	++	+

*EMT* epithelial mesenchymal transition, *CSC* cancer stem cell, *WHA* wound-healing assay, *TMA* traswell migration assay, *SP* side population.

^a^E: Epithelium, I: Intermediate between epithelium and mesenchmal, M: Mesenchyma.

^b^+ : > 30 h of doubling time (DT), ++: 20–30 h of DT, +++: < 20 h of DT.

^c^± : < 40% of wound closure, 40–70% of wound closure, ++: > 70% of wound closure.

^d^ ± : < 60 migration or invasion cells, +: 60–120 maigration or invasion cells, ++ ; > 120 migration or invasion cells.

^e^ ± : < 15 spheres, + : 15–30 spheres, ++; > 30 spheres.

^f^ ± : < 5% SP, + : 5–10% SP, ++; > 10 SP.

^g^ ± : > 1000 nM of IC50, +: 10–1000 nM of IC50, ++: < 10 nM of IC50.

Traditionally, a tumor was considered a group of cells with similar characteristics. However, currently, a tumor tissue is recognized as an assembly of heterogeneous tumor cells with distinct cellular properties, including morphology, proliferative potential, motility, and function. In the present study, we successfully extracted and established various tumor clones with infinite proliferative capacity from canine PAC tissues. Although it is unknown why various cell lines are obtained from one tissue, this variety may reflect the heterogeneity and differentiation potential of tumor cells.

EMT is associated with malignancy and is considered a crucial therapeutic target^14–19,37^. Certain cell lines in the present study showed a mesenchymal-like shape, which is a morphological characteristic of EMT. In the cloned cell lines, CPACd-AE3, CPACr-AH4, CPACr-BC9, and CPACr-DC3 showed mesenchymal-like morphologies and relatively high vimentin expression. In contrast, CPACd-DC10, which had a spindle shape, did not show high vimentin expression, whereas CPACd-AB4, which had an epithelial shape but a relatively wide gap between cells, showed relatively high vimentin expression. According to the migration and invasion analyses, CPACd-AB4, CPACd-CG11, and CPACr-BC9 showed high motility. CPACr-BC9 showed mesenchymal morphology, whereas CPACd-AB4 and CPACd-CG11 showed epithelial morphologies but exhibited relatively high vimentin expression. These findings suggest that EMT may have occurred in these three cell lines and that EMT cannot be conclusively determined solely through cellular morphological analysis. In human tumor cells, partial EMT (Hybrid epithelial/mesenchymal phenotypes), characterized by cells displaying both mesenchymal and epithelial characteristics, is recognized as a part of the EMT process^38^. Therefore, it is plausible that partial, rather than complete, EMT has occurred in CPACd-AB4 and CPACd-CG11. Partial EMT is believed to be regulated by tumor microenvironmental factors^39^ and can enhance invasive properties, generate circulating tumor cells and cancer stem cells, and confer chemoresistance^40^. In human PAC cells, the *GRHL2* gene has been implicated in the maintenance of stable partial EMT^41^, although the detailed mechanisms remain incompletely understood. The cell lines we have established exhibit stable partial EMT, suggesting the potential for elucidating the mechanisms responsible for stabilizing partial EMT and developing therapeutics targeting partial EMT.

CSCs are a subset of tumor cells characterized by self-renewal, differentiation capacity, and chemoresistance. These properties make CSCs crucial targets for tumor therapy^18,21,22^. In the present study, we assessed CSC properties using two different methods: sphere-formation and SP assays with DCV. CPACd-AB4, CPACd-CG11, and CPACr-AD4 showed relatively high sphere-forming ability and SP population; however, in CPACd-AE3, CPACd-DC10, CPACr-AH4, and CPACr-BH12, the results of the two assays were not concordant. Assessing CSC-associated antigens is widely used for detecting CSCs. In human oncology, several antigens are used as CSC markers, including CD44, CD133, CD24, and EpCAM; however, the expression patterns appear to vary with respect to tumor origin^20,22^. Although CD44 and CD133 expression have been previously assessed in a xenograft model of canine PAC, their utility as CSC markers has not been indicated^42^. In human PAC, these two antigens have been reported to have poor diagnostic value in CSC detection^43^. Reliable markers for the detection of CSCs may be determined by further assessment using developed canine PAC cell lines.

EMT and CSCs are closely linked^18,20^. In our study, CPACd-AB4 and CPACd-CG11 showed both EMT and CSC phenotypes. Several transcription factors are associated with the development of EMT and maintenance of CSCs. Transforming growth factor-β (TGF-β) has pleiotropic functions in tumor progression^44^. In epithelial cells, TGF-β acts as a tumor suppressor by inhibiting cell growth and inducing apoptosis. In contrast, in the advanced stages of tumorigenesis, TGF-β induces EMT and stimulates cell proliferation and survival in tumor cells^45^. TGF-β overexpression has been reported to be associated with malignancy in human tumors^46^. In human liver tumor cells, autocrine stimulation of TGF-β was reported to induce EMT^47^. Importantly, EMT stimulated by TGF-β induces the selection and expansion of CSCs^48,49^. Moreover, previous reports have indicated that MAPK and PI3K signaling play important roles in EMT or the maintenance of CSCs by EMT^50–53^. In our study, relatively high phosphorylation of Erk1/2 and expression of vimentin were observed in CPACr-AH4 and CPACr-DC3. However, Erk1/2 phosphorylation did not appear to correlate with cellular motility. CPACd-AB4 induced phosphorylation of Akt at a relatively high level; however, the other cell lines with CSC phenotypes did not show high phosphorylation of Akt. Further assessment of other signals, including the TGF-β signal, is needed to reveal the detailed mechanism of EMT and CSCs in the canine PAC. On the other hand, Andriani et al. demonstrated that the differences in susceptibility to CSCs induction by TGF-β were dependent on the balance between epithelial and mesenchymal features and cells developing partial EMT exhibit the highest sensitivity in human PAC cells^54^. The cell lines we have established, exhibiting varying degrees of EMT, hold significant potential possibilities as models for elucidating the association between CSCs and EMT.

Canine PAC resembles human lung tumors in never-smokers. Adjuvant chemotherapy, including vinorelbine, is indicated for patients with lung cancer. However, in some lung tumors that are intrinsically resistant to chemotherapy, acquired resistance has been shown to develop rapidly even in initial responders^55^. Chemotherapy-resistant cells contribute to relapse. Although few reports are available for canine cases, chemotherapies, including vindesine, mitoxantrone, vinorelbine, and toceranib, are not fully effective^8–10,56,57^. Additionally, the mechanisms of resistance have not been elucidated. In the present study, the sensitivity to vinorelbine differed among the cell lines. CPACr-p and the clones, appeared to be relatively sensitive to vinorelbine, and Ras prenylation levels were lower than that in CPACd-p and cloned cell lines. This finding indicates that Ras signaling may be related to the mechanism that determines susceptibility to vinorelbine. However, Erk1/2 and Akt signaling may not be involved in the mechanisms common to these cell lines. Importantly, cell lines with EMT- and CSC-like phenotypes, such as CPACd-AB4 and CPACd-CG11, were resistant to vinorelbine. This finding indicates that EMT and stemness may contribute to chemoresistance in canine PAC. In contrast, although CPACd-AC12 showed resistance to vinorelbine, it showed poor expression of EMT and CSCs phenotypes. Additional studies using the cell lines we have established may reveal the mechanisms of resistance to vinorelbine, not only in canine but also in human PAC.

The molecular characterization of canine PAC remains limited in comparison to human lung cancer. In human NSCLC, EGFR and HER2 have been shown to be overexpressed and recognized as significant therapeutic targets^29^. Also, in canine PAC, previous reports showed overexpression or activation of RTKs^31–33^, but the utility as therapeutic targets has not been established. In our findings, the established cell lines expressed EGFR and HER2 at various levels. Therefore, some of these cell lines might retain the expression of RTKs, which could be beneficial for the development of targeted therapies focusing on RTKs. On the other hand, in human lung cancer, drug resistance to EGFR inhibitors has also become a significant concern^58^. Due to the high similarity between PAC in humans and dogs, further evaluation of cellular characteristics associated with RTKs of these established cell lines could potentially be applied to the research aimed at overcoming resistance to RTK inhibitors in human lung cancer.

The present study had several limitations. First, we did not perform transplantation in mice. Second, we did not assess the transcription factors associated with EMT, including Zeb, SNAIL, and SLUG^37^. These experiments can possibly further clarify the EMT and CSC phenotypes and the detailed mechanisms underlying these phenotypes. Third, cell lines with various properties may not reflect the heterogeneity in tumor tissue but an in vitro selective pressure during the culture process. However, this possibility is difficult to be excluded. Finally, we have not been able to verify whether the established cell lines represent a general profile of canine pulmonary adenocarcinoma. However, given the absence of a defined general profile for canine PAC, it is difficult to say that. Further comparative investigation using whole-genome analysis and epigenetics methods between the established cell lines and tumor tissues from other dogs may substantiate the representativeness of these cell lines in canine PAC.

In conclusion, we successfully generated novel canine PAC cell lines with various characteristics. Moreover, we assessed the cellular properties in vitro, focusing on the EMT and CSC phenotypes. Our results indicate that EMT and stemness may play a role in resistance to vinorelbine. We also elucidated that the established cell lines exhibit varying levels of expression and activity of RTKs and Ras signaling factors. Therefore, these cell lines will facilitate the development of novel therapeutic strategies targeting EMT, CSCs, and intracellular signaling pathways and elucidation of the contribution of tumor heterogeneity to tumor biology, including chemoresistance, in human and canine PAC.

## Methods

### Tumor sample

A 14-year-old neutered male Maltese dog weighting 4.1 kg presented with a mass in the right posterior lung lobe. The mass was 2 cm in diameter, and metastatic lesions were absent according to diagnostic imaging, including computed tomography. The neoplasm was surgically resected and histopathologically diagnosed as a pulmonary adenocarcinoma (No Boundaries Animal Pathology, LLC, Tokyo, Japan). Further clinical information is described in Supplementary information file. Fresh tissue samples were collected, in accordance with the informed written consent of the owner and the approval of the Ethical Committee of Okayama University of Science (Approval No. 2020-0004), and the methods were performed in accordance with the relevant guidelines and regulations of Okayama University of Science and ARRIVE guidelines.

### Primary culture

Tumor tissues were washed with saline and minced (1 mm). The tumor fragments were disaggregated with dispase (2000 PU/mL; Godo Shusei, Tokyo, Japan) at 37 °C for 5 h with continuous agitation. After washing with phosphate buffered saline (PBS), the digested tissue was resuspended in DMEM (08,458-16, Nacalai Tesque, Kyoto, Japan) or RPMI-1640 (30,264-56, Nacalai Tesque), which are usually used in culture of various tumor cells as basic media, containing 10% deactivated fetal bovine serum (FBS; Biosera, Nuaille, France), 100 U/mL penicillin (Nacalai Tesque), and 100 µg/mL streptomycin (Nacalai Tesque). Cell suspensions were maintained at 37 °C in a humidified 5% CO_2_ incubator. Once the cell culture reached 80–90% confluence, they were repeatedly sub-cultured with 0.25% trypsin solution at a 1:4 dilution. The cell lines were named CPACd-p and CPACr-p and were cultured in DMEM- or RPMI-1640-based media, respectively. The cell lines were maintained for more than 50 passages.

### One cell cloning

The cell lines were cloned from CPACd-p and CPACr-p cells after 20 passages using the limiting dilution method^59^. Cells were seeded at 500 cells/well in a 24-well plate and maintained until 90% confluence under the conditions described above. The cells were then seeded at a density of 1 cell/well in a 96-well plate (384 wells). The plates were examined under a microscope immediately after dispensing the cells and culturing. Wells suspected of containing more than one cell or multiple colonies were not cultured. After 30 passages, based on cellular morphology and subculture intervals, seven lines, which were realistic in terms of number of analyses, were selected from the cloned cell lines derived from CPACd-p and CPACr-p. These cell lines, including the parent cell lines, tested negative for mycoplasma contamination (TaKaRa PCR Mycoplasma Detection Set; Takara Bio Inc., Shiga, Japan).

### Morphologic analysis

Microcover glasses (Matsunami Glass, Osaka, Japan) were placed in the wells of a 12-well plate. The glasses were coated with type I collagen (10 µg/mL; Nippi Company, Tokyo, Japan). The cells were then seeded and cultured in a plate at a density of 1 × 10^5^ cells/well on microcover glasses. At 80–90% confluence, the cells were fixed with methanol and stained with Wright–Giemsa stain (MUTO Pure Chemicals, Tokyo, Japan). The glasses were captured under a light microscope at 40 × and 400 × magnifications. Each experiment was repeated at least thrice.

### Growth curve and measuring of doubling time

The cells were seeded at 1.0 × 10^4^ cells/well in a 24-well plate. After 24, 48, 72, and 96 h, the viable cells were counted using a hemocytometer with 0.5% trypan blue solution. Doubling time was calculated during the logarithmic growth phase (24–96 h) according to the following formula.

$$Doubling\,time (h)= \frac{72\times \mathrm{log}2}{\mathrm{log }\left(the\,number\,of\,cells\,at\,96 h\right)-\mathrm{log} (the\,number\,of\,cells\,at\,24 h)}$$

Each experiment was performed in triplicate and repeated at least thrice.

### Immunoblotting

In addition to the samples from the CPAC cell lines, protein samples from a canine mammary gland tumor cell line, CHMp-13a^60^, and a canine hemangiosarcoma cell line, Re12^61^, were also utilized as epithelial and mesenchymal controls, respectively. CHMp-13a and Re12 were kindly provided by Dr. T. Nakagawa (The University of Tokyo, Japan) and Dr. H. Sakai (Gifu University, Japan), respectively. The results of CHMp-13a and Re12 were exclusively presented in the Supplementary Information file. Cells were lysed with NP40 lysis buffer [1% NP40, 10 mM Tris HCl (pH 7.5), 150 mM NaCl, and 1 mM ethylenediaminetetraacetic acid (EDTA)] supplemented with an EDTA-free protease inhibitor cocktail (Nacalai Tesque) and a phosphatase inhibitor cocktail (Nacalai Tesque). Twenty micrograms of protein were subjected to sodium dodecyl sulfate–polyacrylamide gel electrophoresis (SDS-PAGE) and transferred onto polyvinylidene fluoride (PVDF) membranes (ClearTrans SP, 0.2 µm; Wako Pure Chemical, Osaka, Japan). The membranes were blocked for 1 h with 5% low-fat milk in Tris-buffered saline [TBS; 50 mM Tris–HCl (pH 7.5) and 150 mM NaCl] containing 0.01% Tween 20 and then incubated with each primary antibody overnight at 4 °C, followed by incubation with HRP-conjugated secondary antibody for 1 h at 24 °C. The primary and secondary antibodies are listed in Supplementary Table S2. The blots were developed using SuperSignal West Pico chemiluminescent substrate (Thermo Fisher Scientific, Waltham, Massachusetts) and visualized using Amersham Image 680 (Cytiva, Tokyo, Japan). Each experiment was repeated at least thrice.

### Wound-healing assay

To evaluate the migratory ability of each cell line, a wound-healing scratch assay was performed. The cells were seeded at 3.0 × 10^5^ cells/well in a 24-well plate and cultured. After reaching 100% confluence, the cell monolayers were manually scratched with a sterile 200 µL pipette tip and washed with PBS to remove suspended cells. The adherent cells were cultured for 12 h. Cell migration was observed and captured under a light microscope at 40 × magnification. The migration ability was calculated relative to a gap area at 0 h. Each experiment was performed in triplicate and repeated at least thrice.

### Matrigel invasion assay

To evaluate the migration and invasion abilities of each cell line, a Boyden chamber assay was performed. Culture inserts with 8 µm pore size (Corning, NY) were set on a 24-well companion plate (Corning). Matrigel (200 µL/well; 500 µg/mL; Corning) was added to the upper chamber and incubated for 2 h at 37 °C. Subsequently, the wells were washed with PBS. Wells without the Matrigel layer were used to count the migrating cells. A cell suspension containing 2.5 × 10^5^ cells in 500 µL of serum-free media was added to the upper chamber. Seven hundred and fifty microliters of medium supplemented with 10% FBS were added to the lower chamber. After 20 h of incubation, non-migrating cells on the upper surface of the membrane were removed using a cotton swab. The inserts were fixed and stained with methanol and Wright–Giemsa stain (MUTO Pure Chemicals). The number of migrating and invading cells was counted under a light microscope at 400 × magnification. Each experiment was performed in triplicate and repeated at least thrice.

### Sphere-formation assay

To evaluate the self-renewal activity of each cell line, a sphere-formation assay was performed using the protocol described in a previous report with a slight modification^62^. Cells were seeded at 2000 cells/mL in an ultra-low attachment 24-well plate (Corning). These cells were cultured in serum-free DMEM/F12 (Nacalai Tesque) supplemented with bovine serum albumin (Nacalai Tesque), 5 mM HEPES (Nacalai Tesque), 100 U/mL penicillin (Nacalai Tesque), 100 µg/mL streptomycin (Nacalai Tesque), 20 ng/mL human recombinant EGF (Peprotech, NJ), 10 ng/mL human recombinant bFGF (Peprotech), and 1% B27 supplement (Invitrogen, CA). EGF, bFGF, and B27 supplement were used to stabilise the formation and maintenance of sphere. DMEM/F12 medium supplemented with 2 ng/mL EGF and 1 ng/mL bFGF was added to the culture every other day for 10 days. Spheres were counted under a light microscope at 40 × magnification. Each experiment was performed in triplicate and repeated at least thrice.

### Identification of side population (SP)

To detect the side population of each cell line, the cells were stained with DCV (Thermo Fisher Scientific) and analyzed by flow cytometer. Cells (5 × 10^5^) were labeled with 5 µM DCV alone or in combination with 50 µM verapamil (Sigma-Aldrich, St. Louis, Missouri). After 90 min of incubation at 37 °C, the labeled cells were washed with ice-cold PBS three times and analyzed by flow cytometer (LSRFortessa X-20; BD Biosciences, Franklin Lakes, NJ) and FlowJo software version v10.8.1 (Tree Star, Ashland, OR). Each experiment was repeated at least thrice.

### Cell-proliferation assay

To evaluate the antiproliferative effect of vinorelbine on each cell line, a WST-8 assay was performed. Cells were seeded at 0.5 × 10^4^ cells/well and treated with different doses of vinorelbine (1 nM–10 µM; Wako Pure Chemical) for 48 h. Subsequently, cell counting kit-8 (10 µL/well; Dojindo, Kumamoto, Japan) was added. After 2 h of incubation, the absorbance was determined at 450 nm using an SH-1300 microplate reader (Corona Electric, Ibaraki, Japan). The percentage of absorbance was calculated against the absorbance for untreated cells (control). Each experiment was performed in triplicate and repeated at least thrice.

### Statistics

All data are presented as the mean ± standard deviation (SD). The normality and homogeneity of variances were verified using the Shapiro–Wilk and Bartlett's tests, respectively. Statistical comparisons between the CPACd-p and CPACr-p cell lines were performed using the unpaired Student’s t-test. Statistical comparisons between the parent and cloned cell lines were performed by one-way ANOVA with Dunnett’s multiple comparison post hoc test using Bell Curve for Excel (Social Survey Research Information Co. Ltd, Tokyo, Japan). Differences were considered statistically significant at *P* < 0.05.

### Supplementary Information

Supplementary Information.

## Data Availability

The datasets used in the present study are available from the corresponding author upon reasonable request.
