# Supplementary material for: Phenotypic and molecular characterization of novel pulmonary adenocarcinoma cell lines established from a dog
Source: Sci Rep. 2023 Oct 5;13:16823. doi: 10.1038/s41598-023-44062-1 (PMC10556002; doi:10.1038/s41598-023-44062-1)

Phenotypic and Molecular Characterization of Novel Pulmonary Adenocarcinoma Cell Lines Established from a Dog

Kosuke Kobayashi*, Reika Deja Takemura, Jiro Miyamae, Ikki Mitsui, Kohei Murakami, Kenji Kutara, Kohei Saeki, Teppei Kanda, Yasuhiko Okamura, Akihiko Sugiyama

Faculty of Veterinary Medicine, Okayama University of Science, Ikoino-oka 1-3, Imabari Ehime, Japan.

Supplementary Methods

Clinical characteristics of the case

The clinical and histopathological features of the canine pulmonary adenocarcinoma (PAC) case are presented in Supplementary Figure S8. The case initially presented with chronic cough and fainting, leading to a referral to the Okayama University of Science Veterinary Teaching Hospital for further evaluation and treatment of a lung mass. Prior to the hospital visit, the case had been diagnosed with myxomatous mitral valve disease (ACVIM stage B2), bradyarrhythmia, and lymphoplasmacytic gastroenteritis. The patient had been receiving various medications, including alacepril, pimobendane, amlodipine, cilostazol, spironolactone, omeprazole, and mosapride citrate. A preoperative CT scan revealed a tumor mass with a diameter of 2 cm in the posterior lobe of the left lung, with no evidence of metastasis (T1N0M0, stage I). The lung mass was completely resectable through surgery.

In the histopathological examination, the following findings were observed: A well-demarcated mass was identified in the left caudal lobe, characterized by tubulopapillary proliferation of tall columnar epithelial cells exhibiting mild nuclear atypia. The tumor cells displayed distinct cell boundaries, a moderate to abundant eosinophilic cytoplasm, mildly anisokaryotic ovoid vesicular nucleus, and one to a few distinct nucleoli. There were 24 mitoses observed in 10 high-power fields (equivalent to 2.37 mm^2). In some regions of the mass, the tumor cells exhibited mild loss of cellular polarity. The tumor interstitium consisted of scant fibrous connective tissue, and scattered necrotic foci were present within the tumor. Additionally, a large cavity containing necrotic debris, red blood cells, macrophages, and neutrophils was observed at the center of the mass. No evidence of tumor invasion was noted in the lymphatics and blood vessels within the specimen. The excision of the tumor was deemed complete.

Postoperative adjuvant therapy, including chemotherapy, was not administered. To date, there has been no recurrence in the lungs. However, one year and 11 months after surgery, a mass was discovered in the ascending colon. It remains unclear whether this lesion was a metastasis of PAC, as no biopsy was conducted. At the time of writing (approximately 1000 days post-surgery), the patient had not experienced serious clinical symptoms and was alive.

Supplementary Figure Legends

Supplementary Figure S1. Phase contrast micrographs of the canine PAC cell lines. Left and right panels in every figure (a-p) are images captured under a light microscope at 40× and 400× magnification, respectively. Scale bar: black, 500 µm; white, 50 µm.

Supplementary Figure S2. Cell proliferative capacity in the canine PAC cell lines. Cell growth curve of the parent cell lines and cloned cell lines of (a) CPACd and (b) CPACr are represented. Each cell lines were seeded at 1.0×10^4 cells/well. Cells were trypsinized and counted using a hemocytometer and trypan blue every 24 h. Data are represented as the mean ± SD at each time point. Results are representative of three independent experiments. (c) Doubling times in each cell line were calculated. Results are represented as the mean ± SD of three independent experiments. $^*P<0.05$, $^{**}P<0.01$: The doubling time is significantly shorter than the parent cell lines; †† $P<0.01$: The doubling time is significantly longer than the parent cell lines.

Supplementary Figure S3. Representative images of wound-healing assay. Cell monolayers (100 % confluent) were manually scratched with sterile 200 µL pipette tip, and washed with PBS to remove suspended cells. The adherent cells were captured at 0 h (left panel) and 12 h (right panel) under a light microscope at 40× magnification. Results are representative of three independent experiments. Scale bar: 500 µm.

Supplementary Figure S4. Representative images of Matrigel invasion assay. Cells on the lower chamber were imaged after 20 h incubation with (right panel) or without Matrigel layer (left panel: control) under light microscope at 40× magnification. Results are representative of three independent experiments. Scale bar: 500 µm.
Supplementary Figure S5. Invasion index. In the Matrigel invasion assay, the number of migrating (control wells) and invading cells (Matrigel wells) were counted in 4 fields in each well under a light microscope at 400× magnification. The number of invading cells was divided by the average number of migrating cells. Data are represented as the mean ± SD of triplicated wells. Results are representative of three independent experiments. **P<0.01: The invasion/migration (%) is significantly higher than that of the parent cell lines; †† P<0.01: The invasion/migration (%) is significantly lower than that of the parent cell lines.
Supplementary Figure S6. Representative images of spheres. Spheres cultured in the ultra-low attachment condition for 10 days were captured under light microscope at 40× magnification. Results are representative of three independent experiments. Scale bar: 500 µm.
Supplementary Figure S7. Dot plots of cells stained with Vybrant DyeCycle Violet (DCV). Each cell line (5×10^5 cells/500 µL) was treated with (right panel) or without (left panel) verapamil (50 µM) and stained with DCV (5 µM). Events were counted at least 10,000. Results are representative of three independent experiments.
Supplementary Figure S8. Clinical and histopathological features of the canine PAC case. (a) Tumor mass detected using computed tomography existed at the posterior lobe of the left lung (2.0 cm in diameter; arrow). (b) Gross aspect of the lung neoplasm. (c) Epithelial cells rich in polymorphism were observed in the stamp specimen (Diff-Quik stain). (d) Histological examination (hematoxylin eosin stain) revealed that neoplastic epithelial cells proliferated forming luminal structures (d-a). However, in the center of the tumor tissue, tumor cells with low differentiation proliferated in fullness (d-b). The interstitial component in the tissue was small.
Supplementary Table S1. IC50 of vinorelbine in canine PAC cell lines.
Supplementary Table S2. Primary and secondary antibodies used for immunoblotting.
Supplementary Figures for immunoblotting. Representative images of immunoblotting. The image contrast and brightness were adjusted using ImageJ software (version 1.53; NIH Image, Bethesda, MD; https://imagej.net/ij/index.html).

Supplementary References

S1. Gómez-Escudero, J., Moreno, V., Martín-Alonso, M., Hernández-Riquer, M.V., Feinberg, T., Colmenar, Á., Calvo, E., Camafeita, E., Martínez, F., Oudhoff, M.J., Weiss, S.J. & Arroyo, A.G. E-cadherin cleavage by MT2-MMP regulates apical junctional signaling and epithelial homeostasis in the intestine. *J. Cell Sci*. **130**, 4013-4027. DOI: https://doi.org/10.1242/jcs.203687 (2017).

S2. Riemann, A., Rauschner, M., Gießelmann, M., Reime, S., Haupt, V. & Thews, O. Extracellular Acidosis Modulates the Expression of Epithelial-Mesenchymal Transition (EMT) Markers and Adhesion of Epithelial and Tumor Cells. *Neoplasia* **21**, 450-458. DOI: https://doi.org/10.1016/j.neo.2019.03.004 (2019).

S3. Fukuoka, H., Cooper, O., Ben-Shlomo, A., Mamelak, A., Ren, S.G., Bruyette, D., Melmed, S. EGFR as a therapeutic target for human, canine, and mouse ACTH-secreting pituitary adenomas. *J. Clin. Invest.* **121**, 4712-4721. DOI: https://doi.org/10.1172/jci60417 (2011).

S4. Clemente-Vicario, F., Alvarez, C.E., Rowell, J.L., Roy, S., London, C.A., Kisseberth, W.C. & Lorch, G. Human Genetic Relevance and Potent Antitumor Activity of Heat Shock Protein 90 Inhibition in Canine Lung Adenocarcinoma Cell Lines. *PLoS One* **10**, :e0142007. DOI: https://doi.org/10.1371/journal.pone.0142007 (2015).

S5. Kobayashi, K., Baba, K., Kambayashi, S. & Okuda,M. Effect of simvastatin on cell proliferation and Ras activation in canine tumour cells. *Vet.Comp.Oncol.* **19,** 99-108. DOI: https://doi.org/10.1111/vco.12644 (2021).

S6. Namba, S., Nakano, R., Kitanaka, T., Kitanaka, N., Nakayama, T. & Sugiya, H. ERK2 and JNK1 contribute to TNF-α-induced IL-8 expression in synovial fibroblasts. *PLoS One* **12**, e0182923. DOI: https://doi.org/10.1371/journal.pone.0182923 (2017).

S7. Lorch, G., Sivaprakasam, K., Zismann, V., Perdigones, N., Contente-Cuomo, T., Nazareno, A., Facista, S., Wong, S., Drenner, K., Liang, W.S., Amann, J.M., Sinicropi-Yao, S.L., Koenig, M.J., La, Perle, K., Whitsett, T.G., Murtaza, M., Trent, J.M., Carbone, D.P. & Hendricks, W.P.D. Identification of Recurrent Activating HER2 Mutations in Primary Canine Pulmonary Adenocarcinoma. *Clin. Cancer Res.* **25**, 5866-5877. DOI: https://doi.org/10.1158/1078-0432.ccr-19-1145 (2019).

Supplementary Figure S1

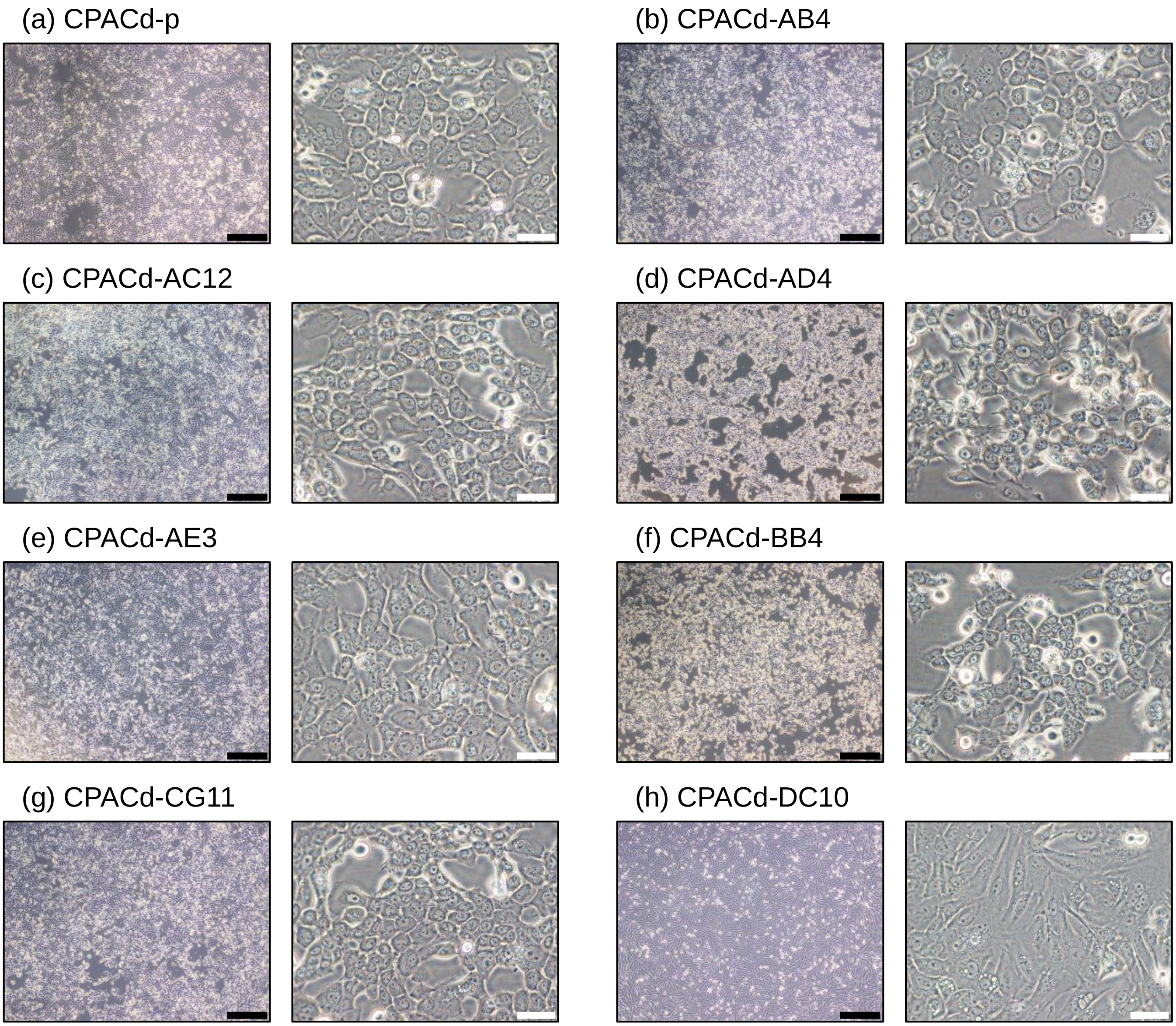

Supplementary Figure S1, continue

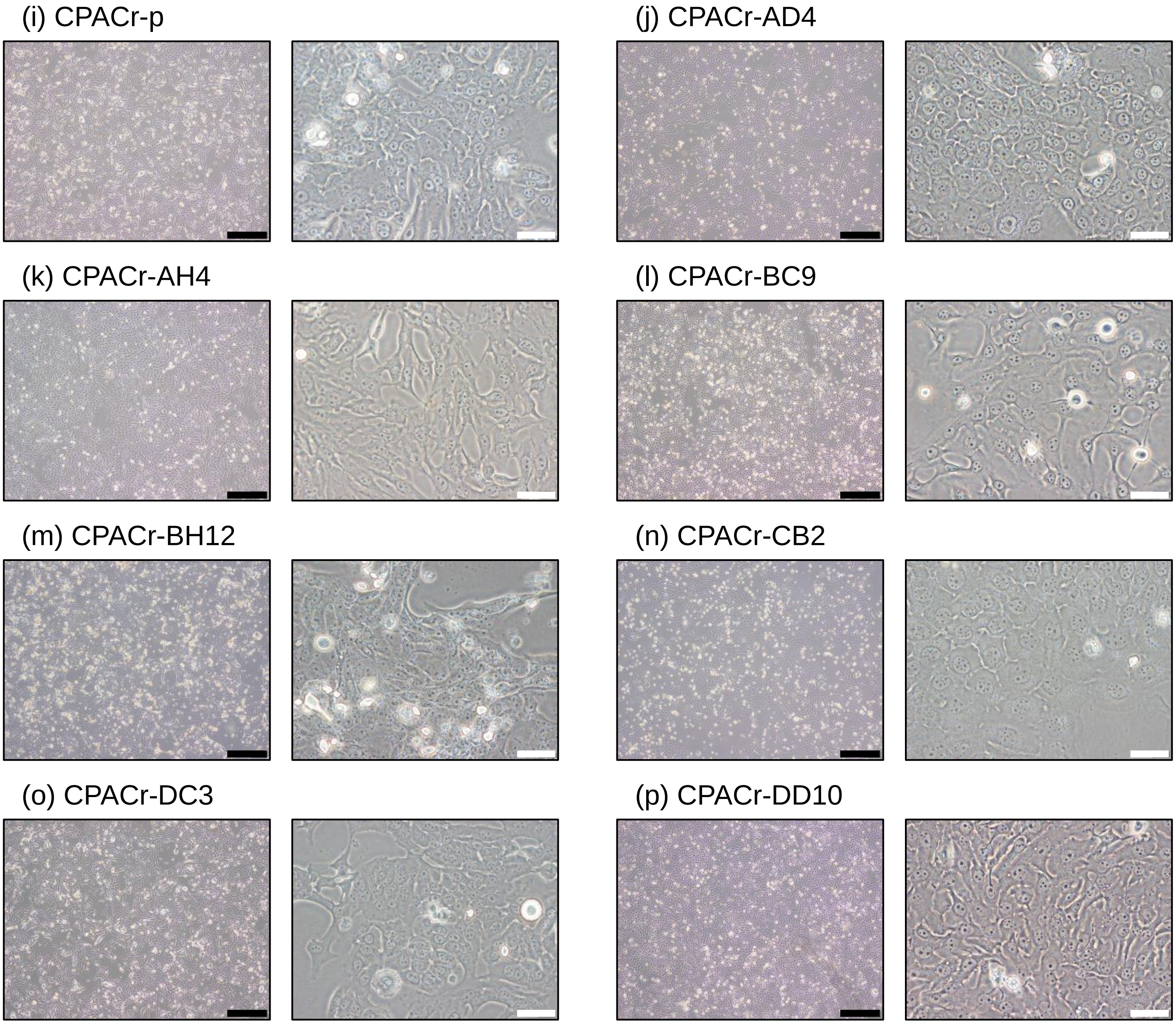

Supplementary Figure S2

(a)

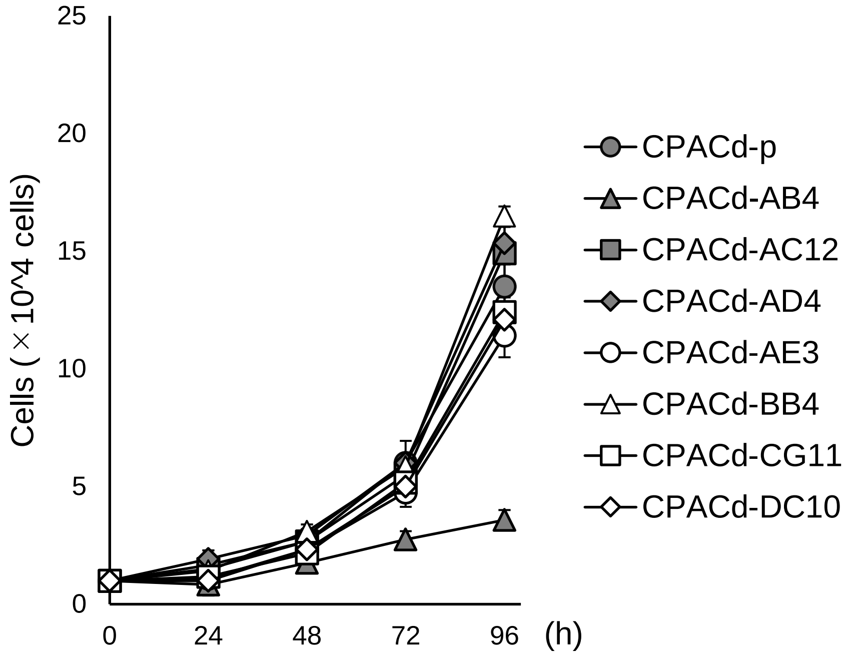

(b)

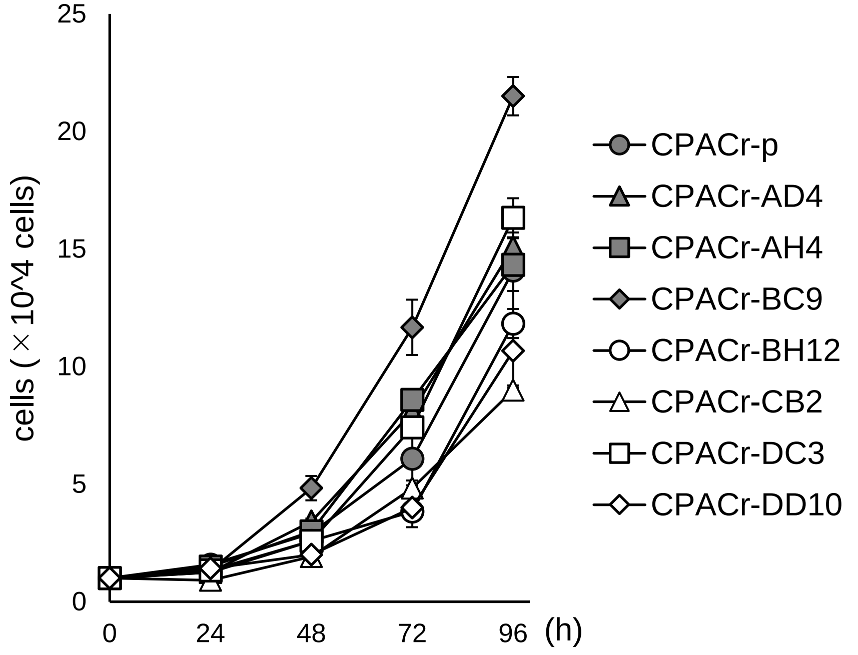

Supplementary Figure S2, continue

(c)

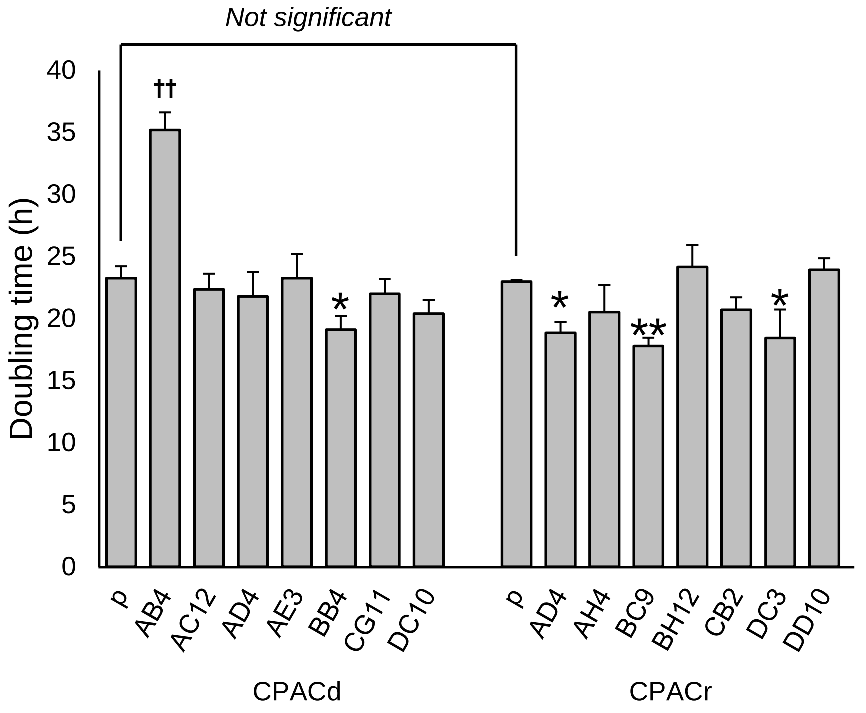

Supplementary Figure S3

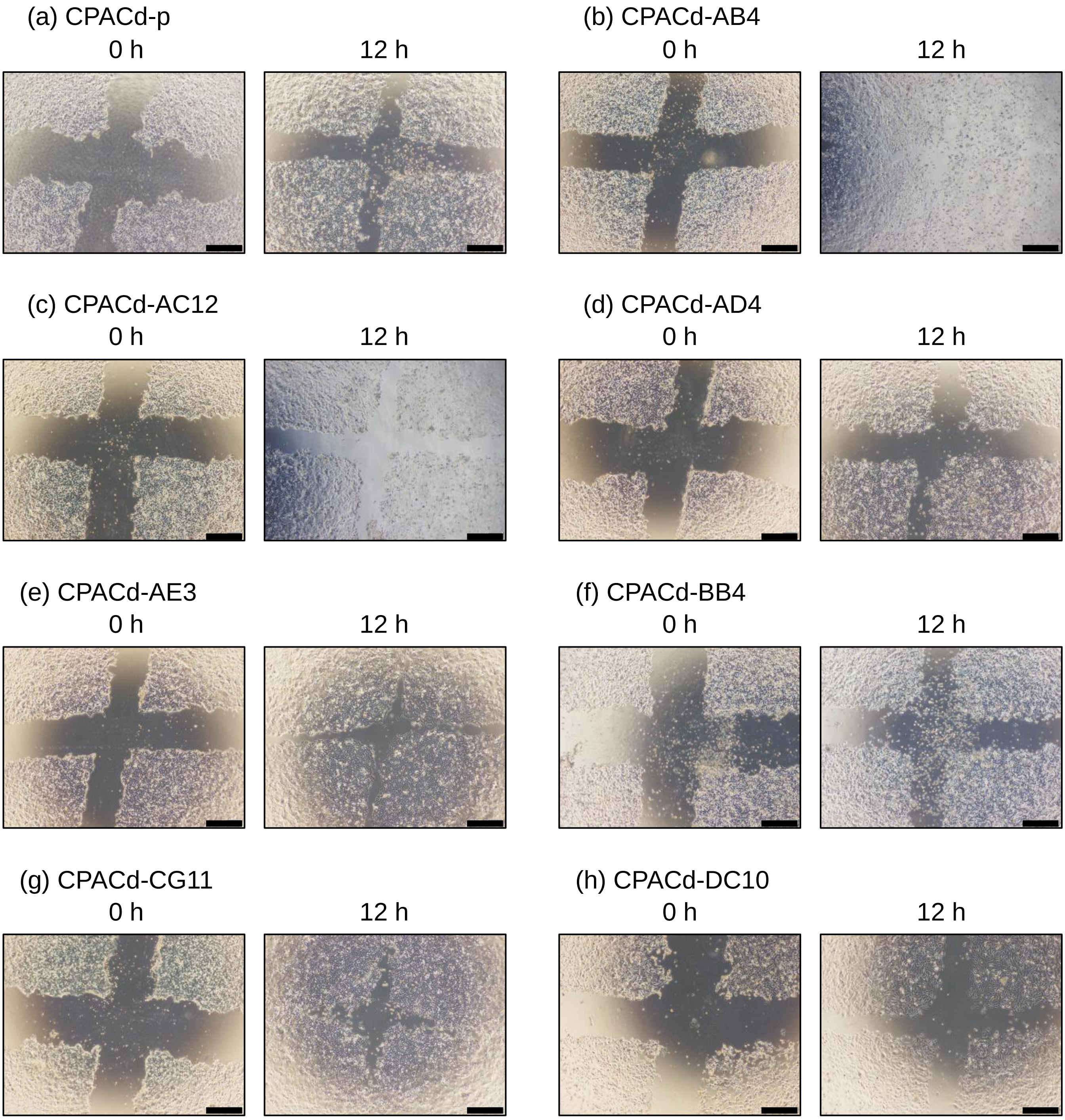

Supplementary Figure S3, continue

(i) CPACr-p

0 h 12 h

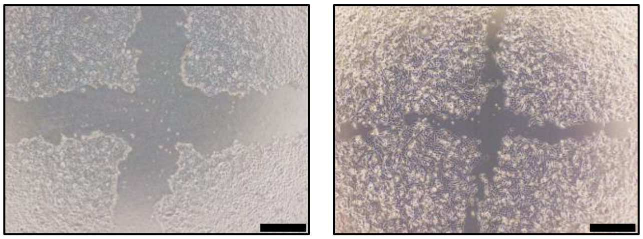

(j) CPACr-AD4

0 h 12 h

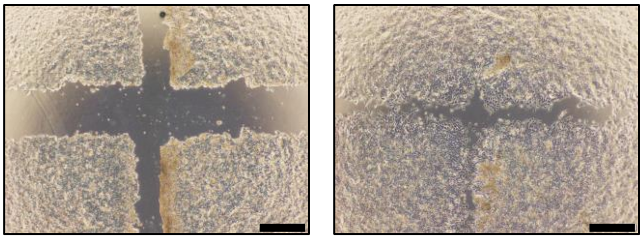

(k) CPACr-AH4

0 h 12 h

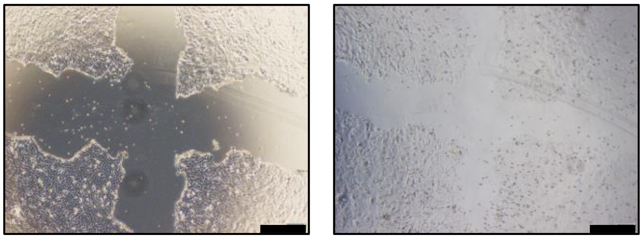

(l) CPACr-BC9

0 h 12 h

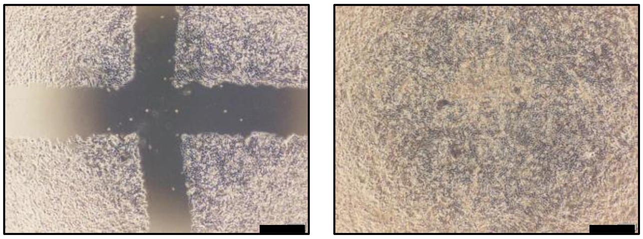

(m) CPACr-BH12

0 h 12 h

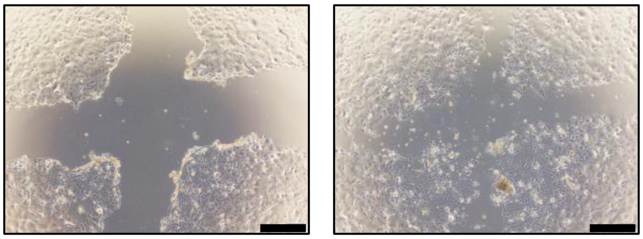

(n) CPACr-CB2

0 h 12 h

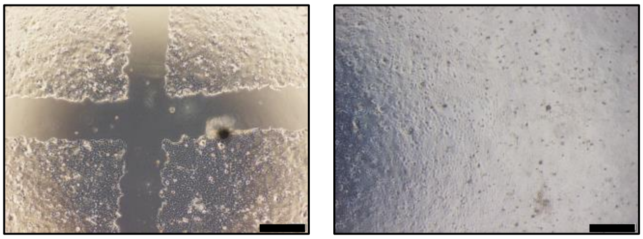

(o) CPACr-DC3

0 h 12 h

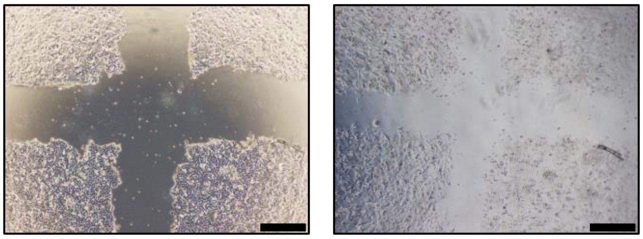

(p) CPACr-DD10

0 h 12 h

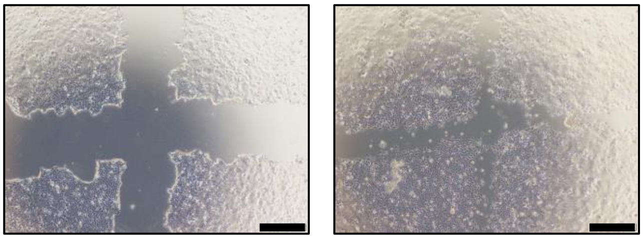

Supplementary Figure S4

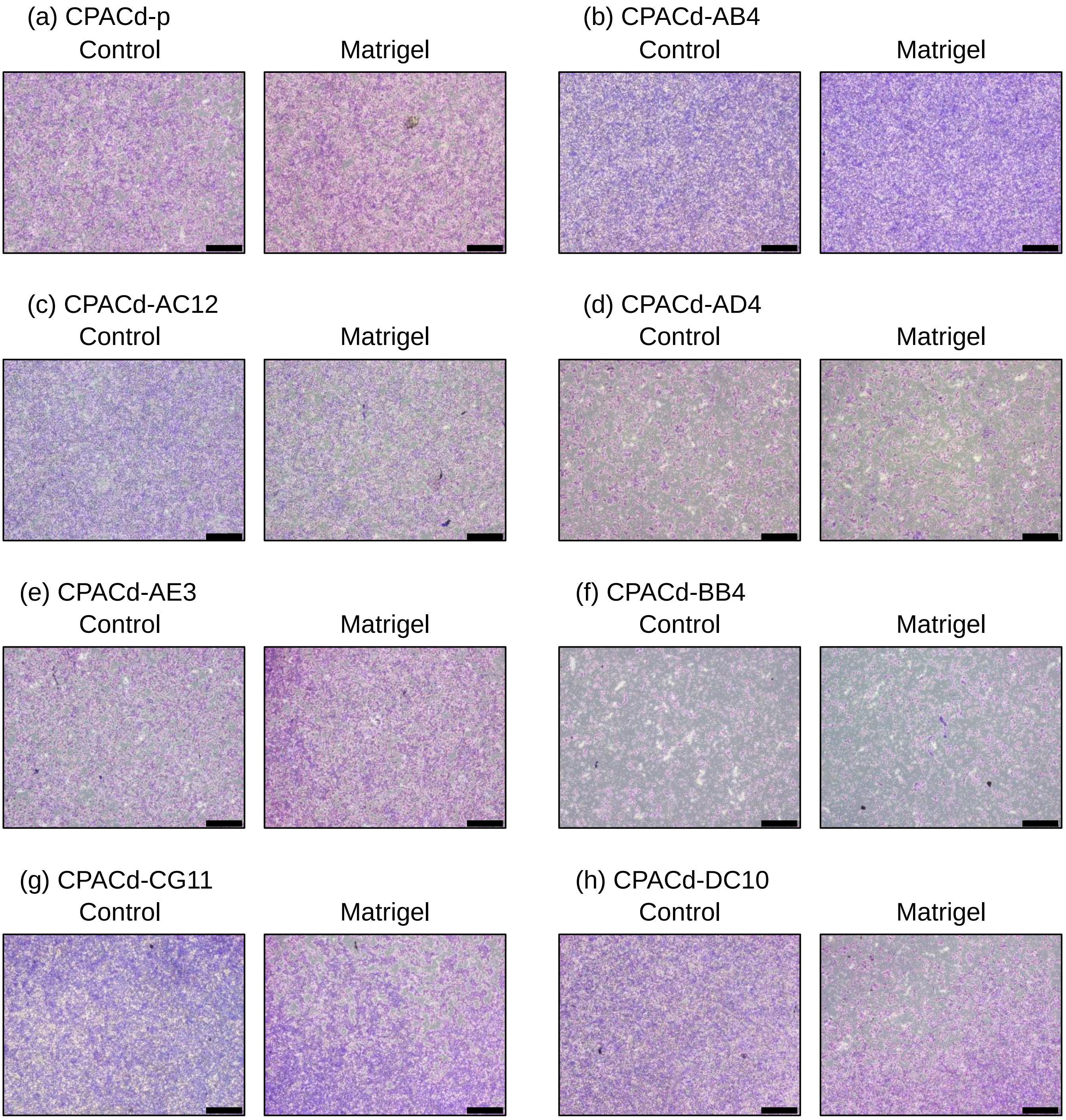

Supplementary Figure S4, continue

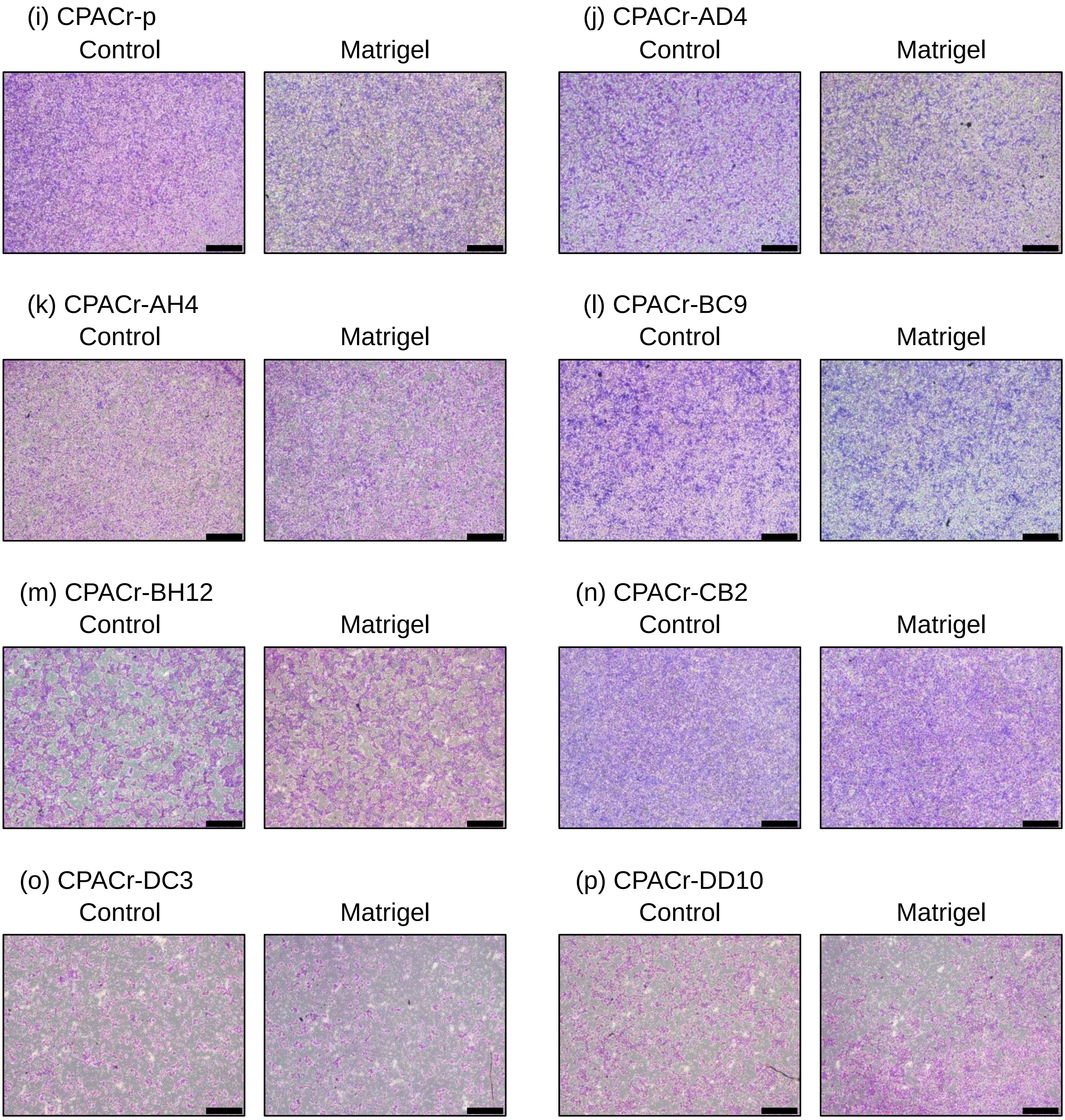

Supplementary Figure S5

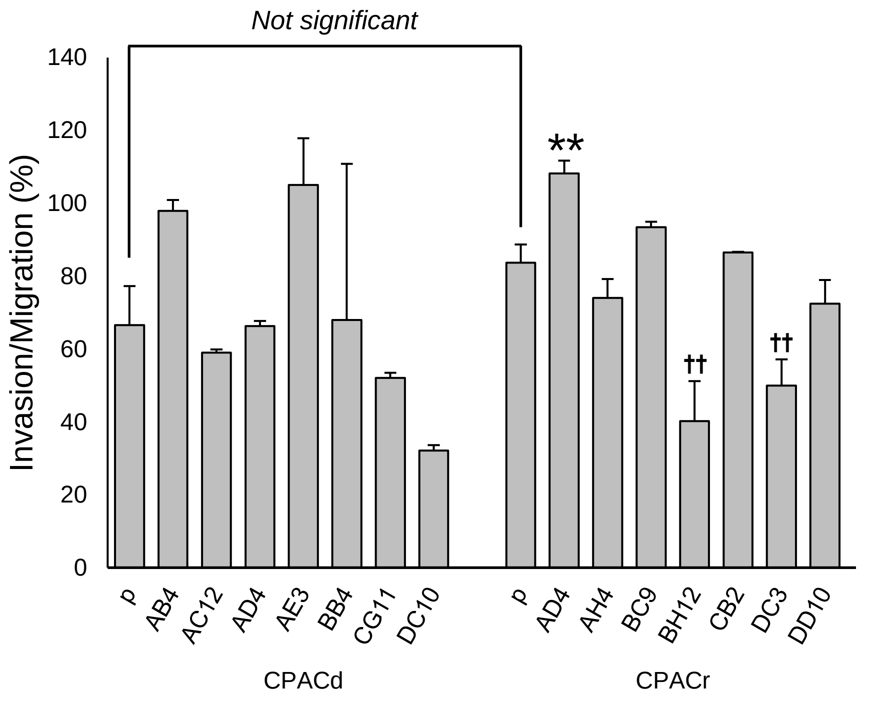

Supplementary Figure S6

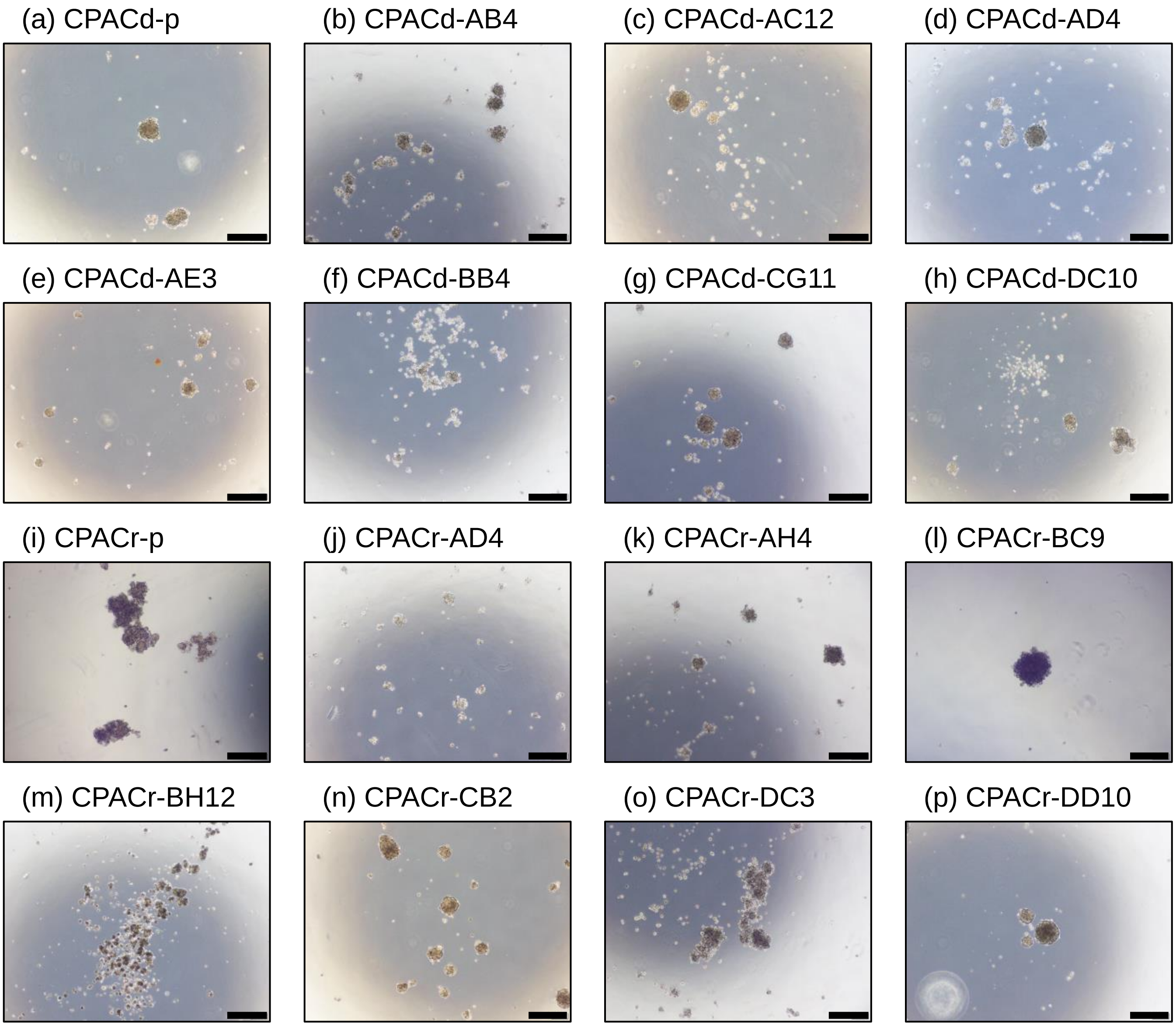

Supplementary Figure S7

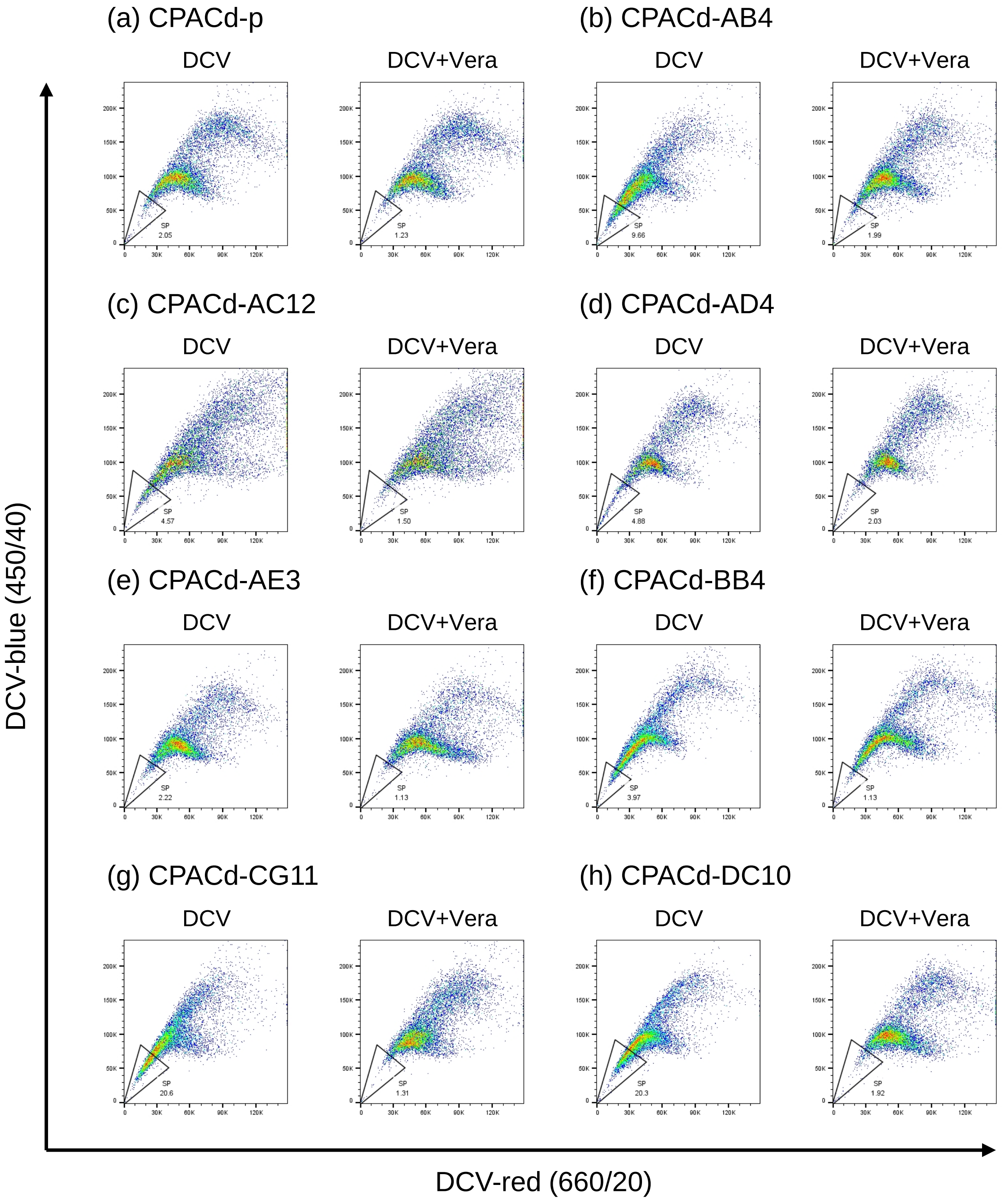

Supplementary Figure S7, continue

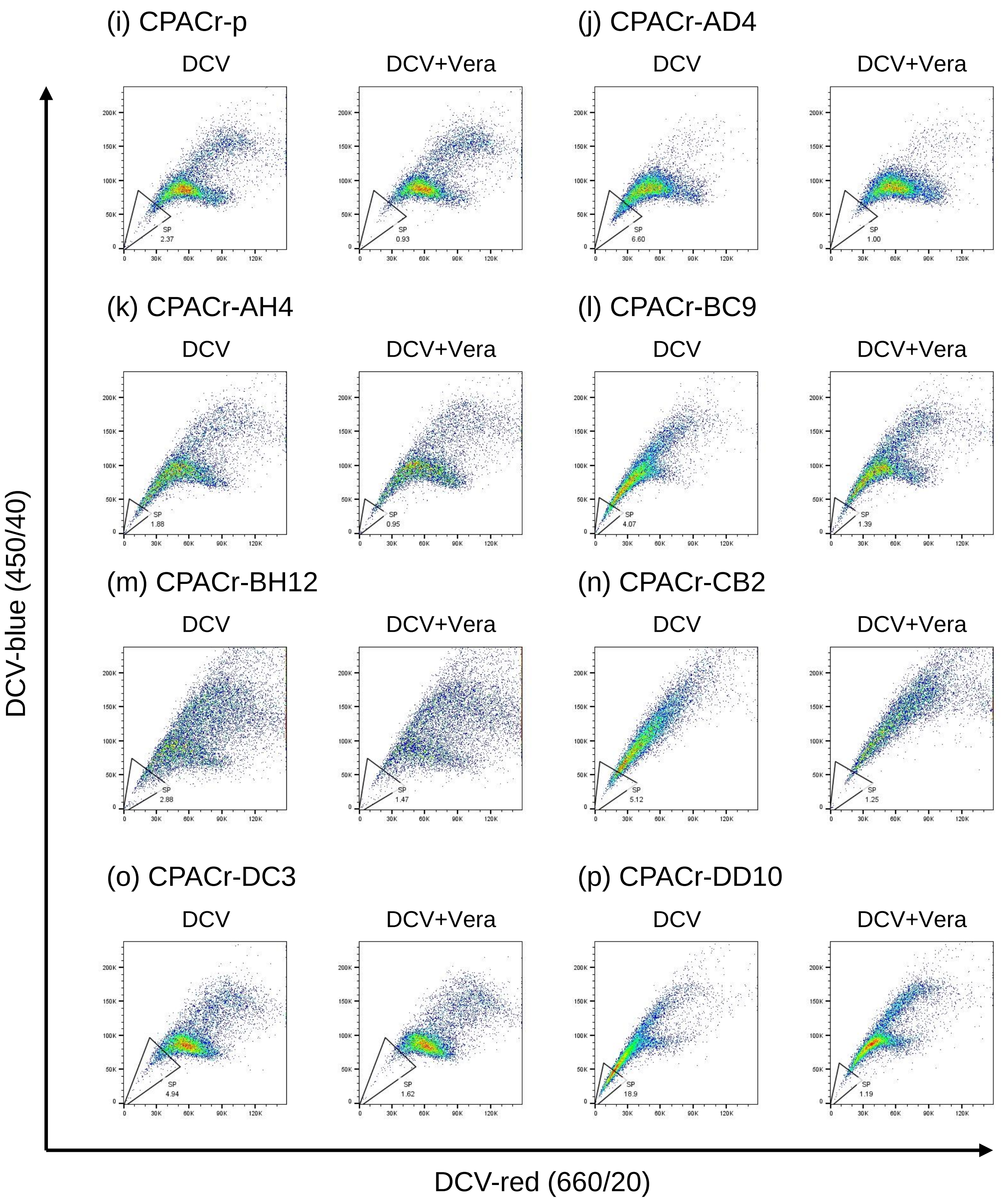

Supplementary Figure S8

(a)

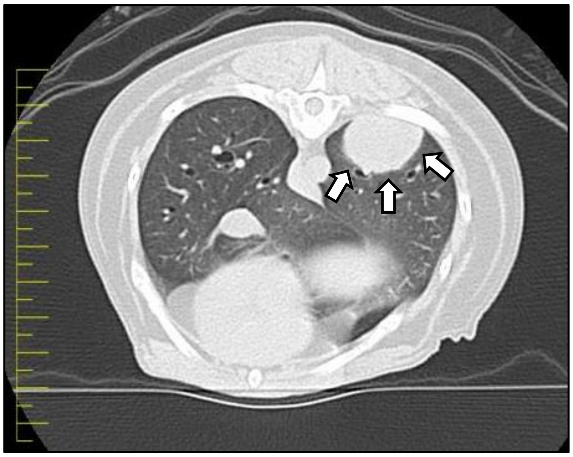

(b)

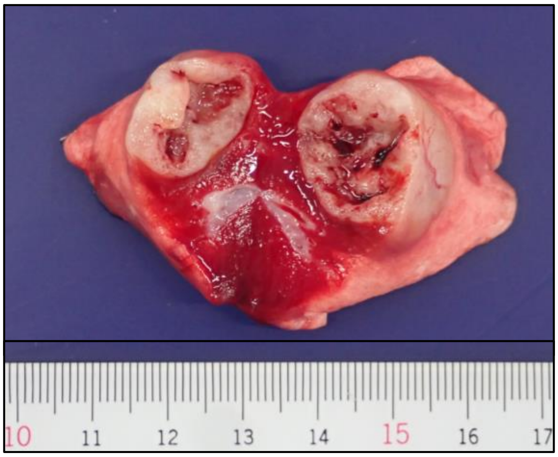

(c)

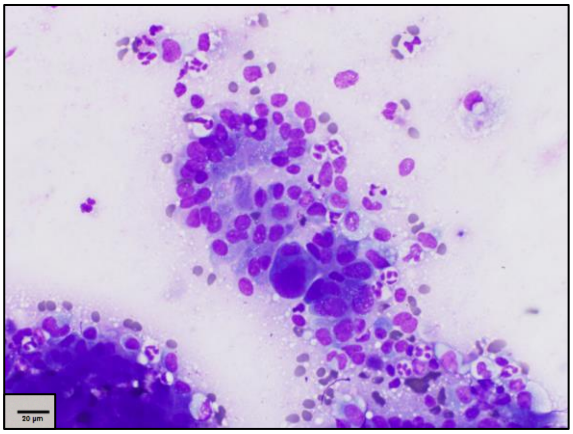

(d)

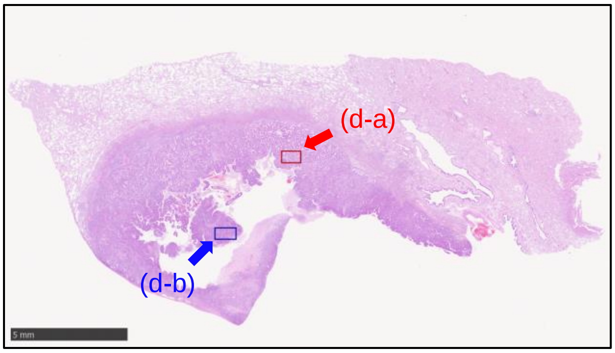

(d-a)

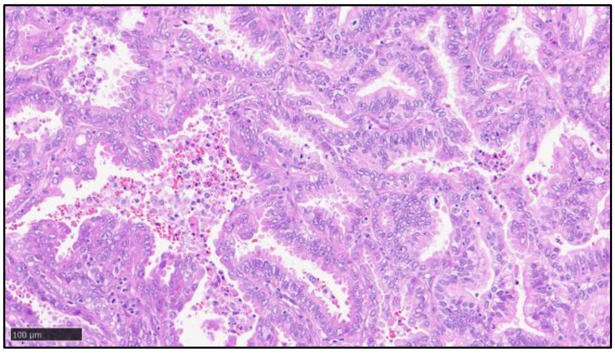

(d-b)

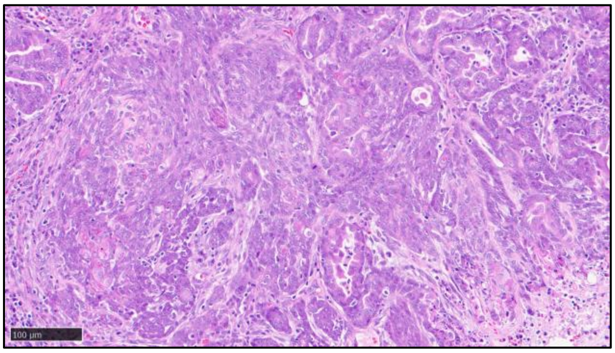

Supplementary Table S1. IC50 of vinorelbine in canine PAC cell lines.

		IC50 (nM)		
CPACd	-p	46.5	±	12.8
	-AB4		> 10000	
	-AC12		> 10000	
	-AD4	24.4	±	17.5
	-AE3	6.8	±	1.6
	-BB4	1.4	±	0.3
	-CG11		> 10000	
	-DC10	61.2	±	15.7
CPACr	-p	1.9	±	0.9
	-AD4	7.6	±	3.5
	-AH4	9.1	±	6.2
	-BC9	10.6	±	3.2
	-BH12	8.0	±	3.3
	-CB2	20.4	±	8.9
	-DC3		< 1	
	-DD10	23.2	±	6.9

Supplementary Table S2. Primary and secondary antibodies used for immunoblotting.

	Target	Conjugate	Manufacture	Catalog number	Clonality	Host	Clone number	Dilution ratio	Refference
Primary	E-cadherin	None	Cell Signaling Technology	3195	Monoclonal	Rabbit	24E10	1:1000	S1
	Vimentin	None	Cell Signaling Technology	5741	Monoclonal	Rabbit	D21H3	1:1000	S2
	EGFR	None	Cell Signaling Technology	2239	Monoclonal	Mouse	1F4	1:1000	S3
	HER2	None	Cell Signaling Technology	4290	Monoclonal	Rabbit	D8F12	1:1000	S4
	Ras	None	Merck	05-516	Monoclonal	Mouse	RAS10	1:1000	S5
	pospho-Erk1/2 (Thr202/Tyr204)	None	Cell Signaling Technology	4370	Monoclonal	Rabbit	D13.14.4E	1:1000	S6
	Erk1/2	None	Cell Signaling Technology	4695	Monoclonal	Rabbit	137F5	1:1000	S6
	phospho-Akt (Ser473)	None	Cell Signaling Technology	4060	Monoclonal	Rabbit	D9E	1:1000	S7
	Akt	None	Cell Signaling Technology	4691	Monoclonal	Rabbit	C67E7	1:1000	S7
	β-actin	None	Sigma	A1978	Monoclonal	Mouse	AC15	1:5000	S5
Secondary	Rabbit IgG H+L	Horseradish Peroxidase	Jacson Immuno Reseach	111-035-003	Polyclonal	Goat	-	1:2000	-
	Mouse IgG H+L			115-035-003	Poluclonal	Goat	-	1:2000	-

Supplementary Figures for Immunoblotting

Immunoblotting images of Figure 2 (a)

#E-cadherin (120 kDa)

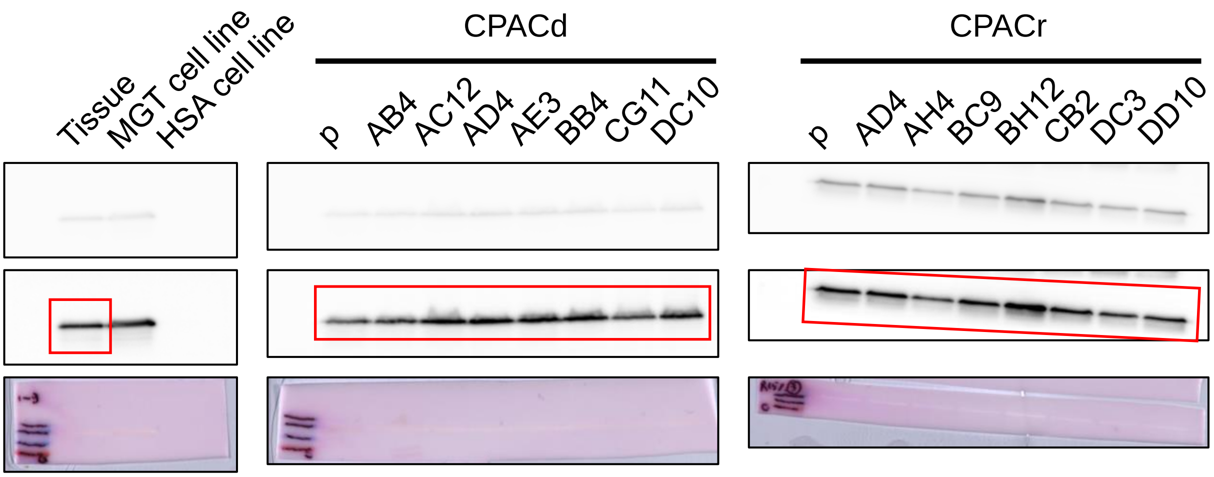

#Vimentin (57 kDa)

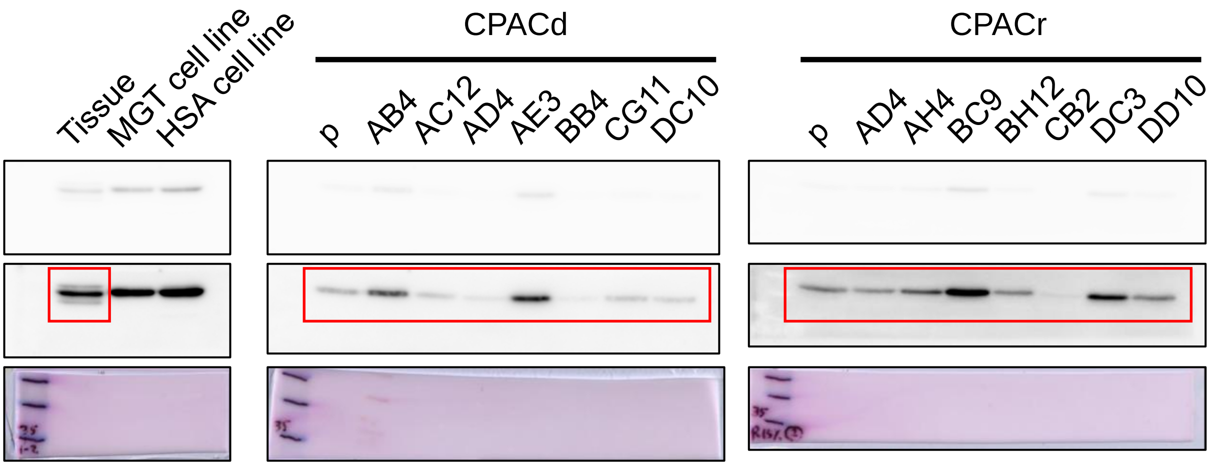

Supplementary Figures for Immunoblotting, continue

Immunoblotting images of Figure 2 (a)

#β-actin (45 kDa)

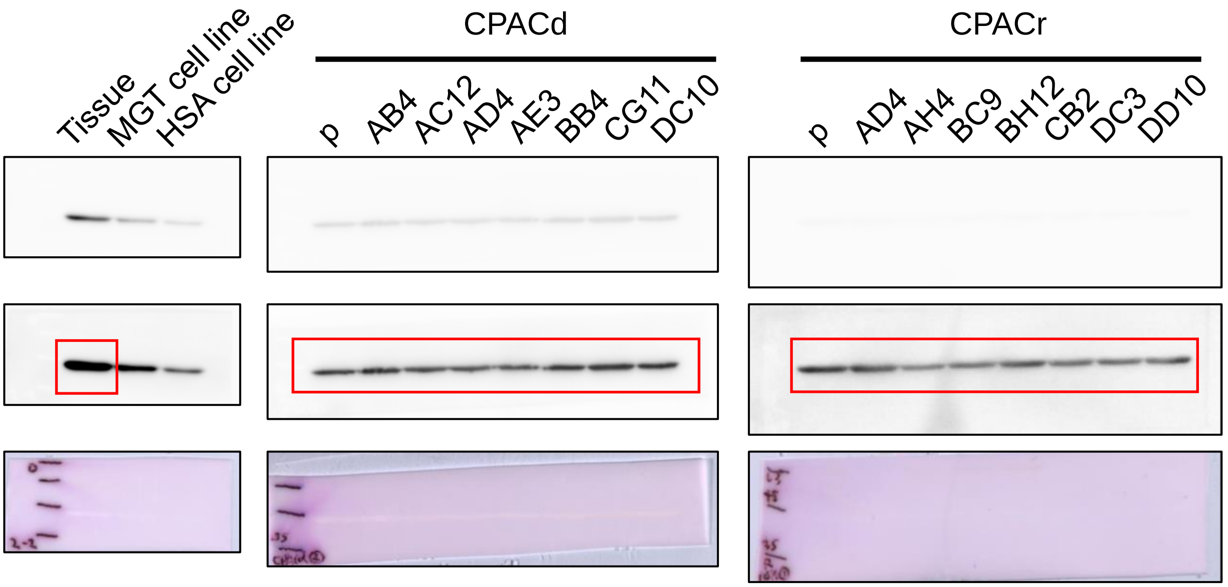

Supplementary Figures for Immunoblotting, continue

Immunoblotting images of Figure 5

#EGFR (175 kDa)

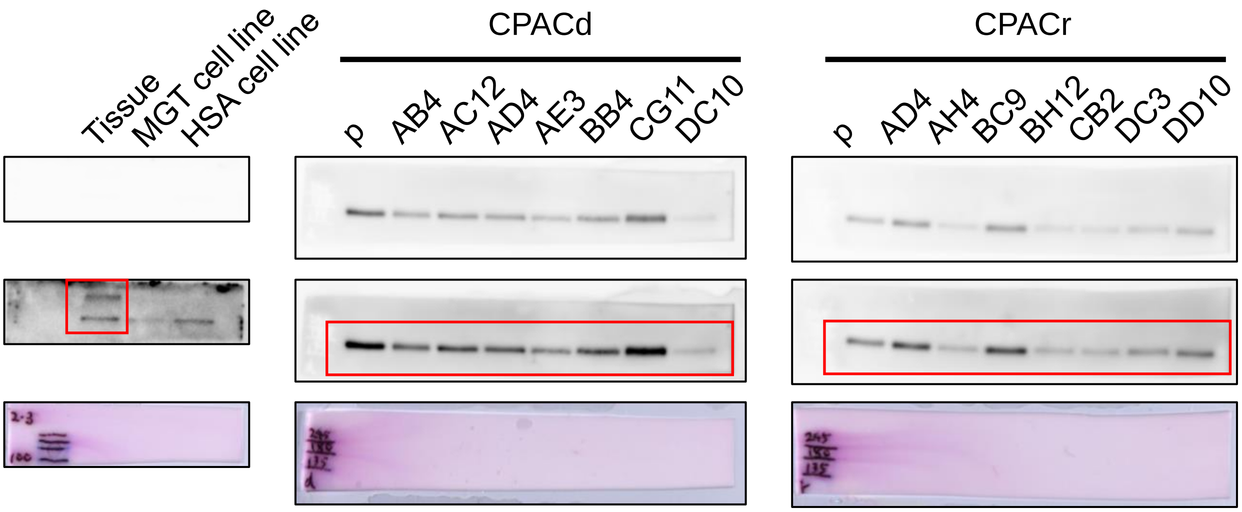

#HER2 (185 kDa)

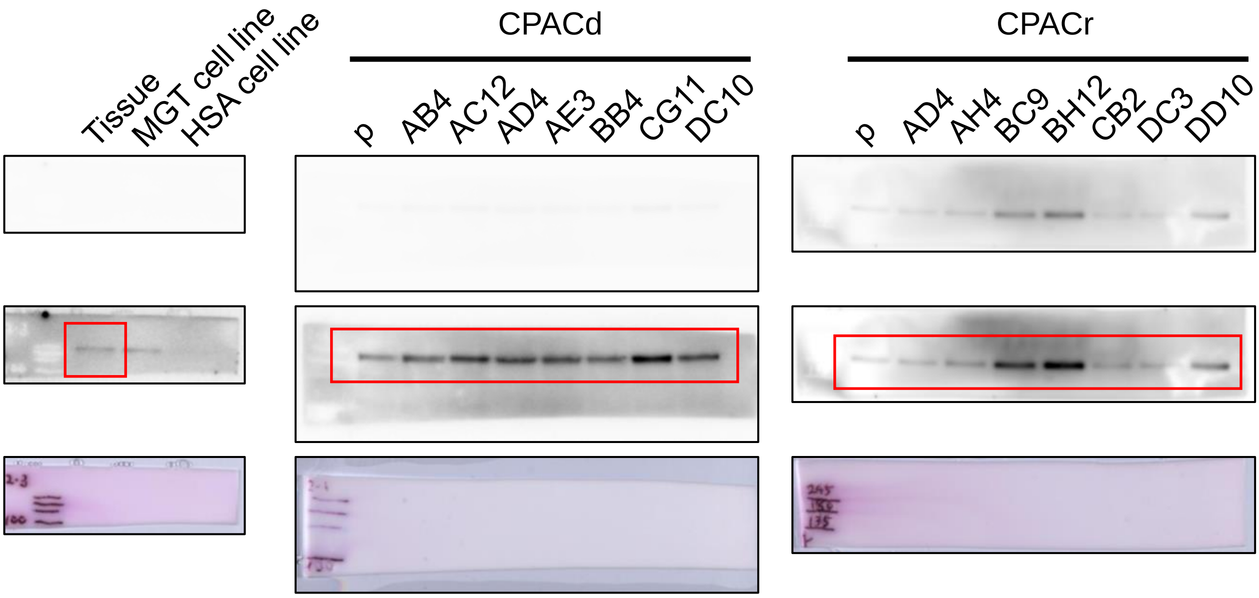

Supplementary Figures for Immunoblotting, continue

Immunoblotting images of Figure 6

#RAS (21 kDa)

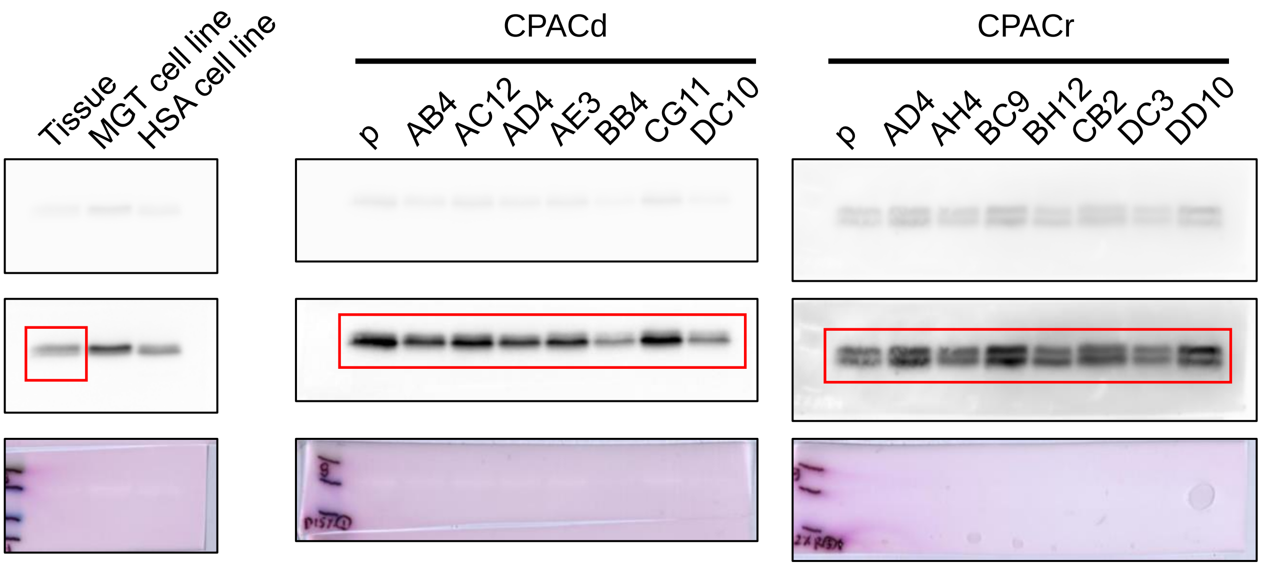

#pErk (42, 44 kDa)

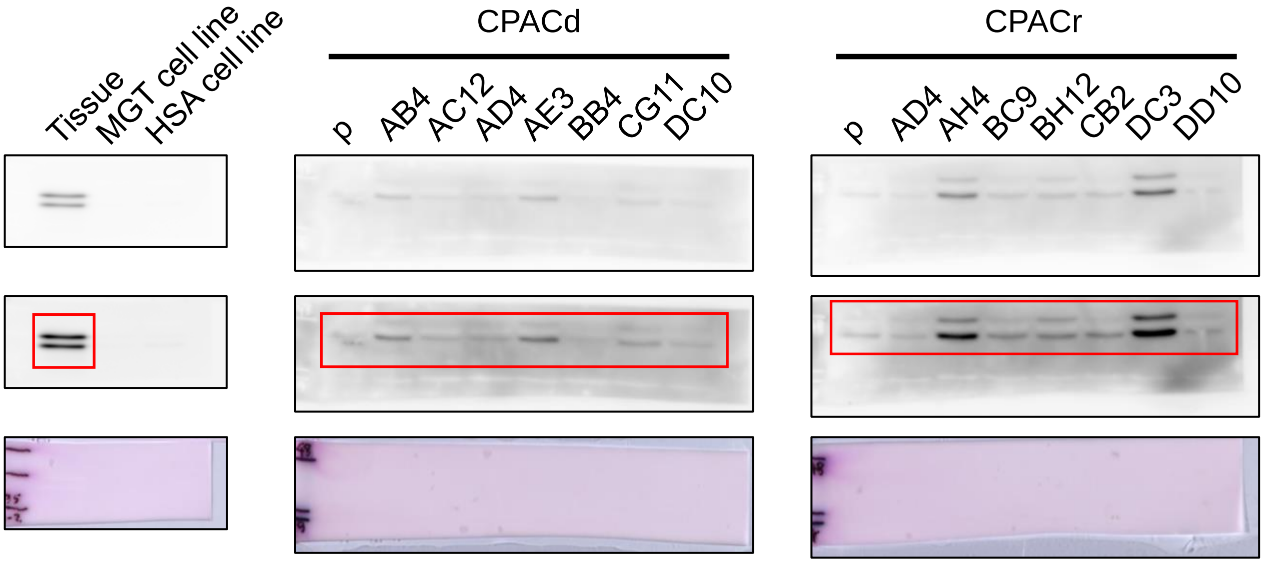

Supplementary Figures for Immunoblotting, continue

Immunoblotting images of Figure 5

#Total-Erk (42, 44 kDa)

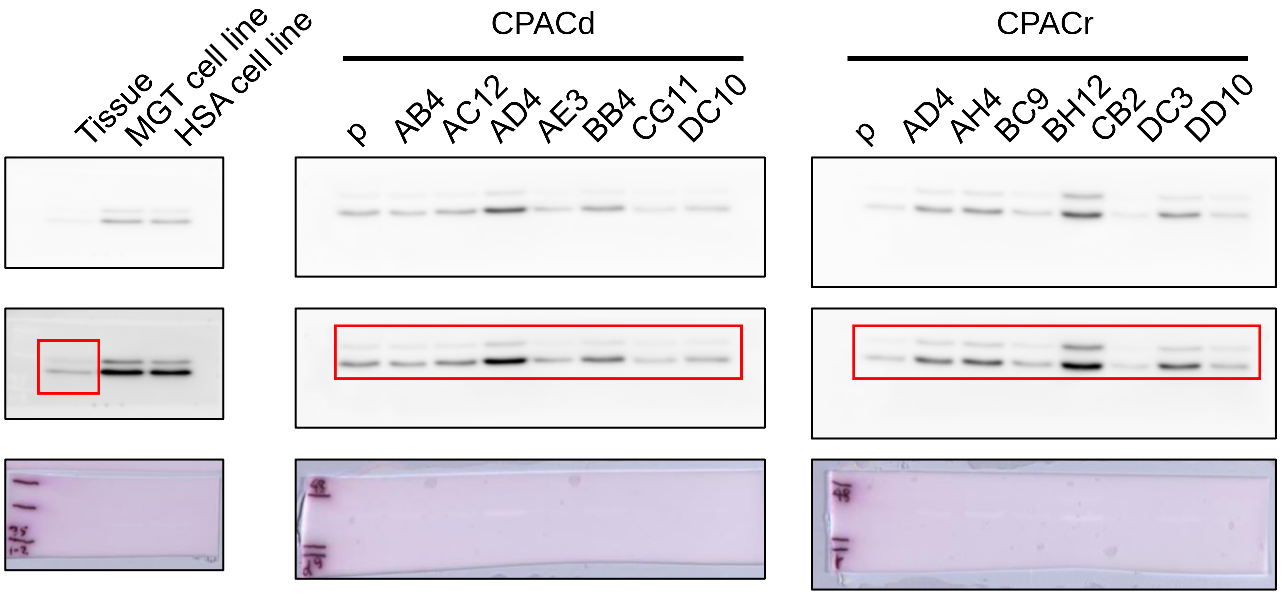

#pAkt (60 kDa)

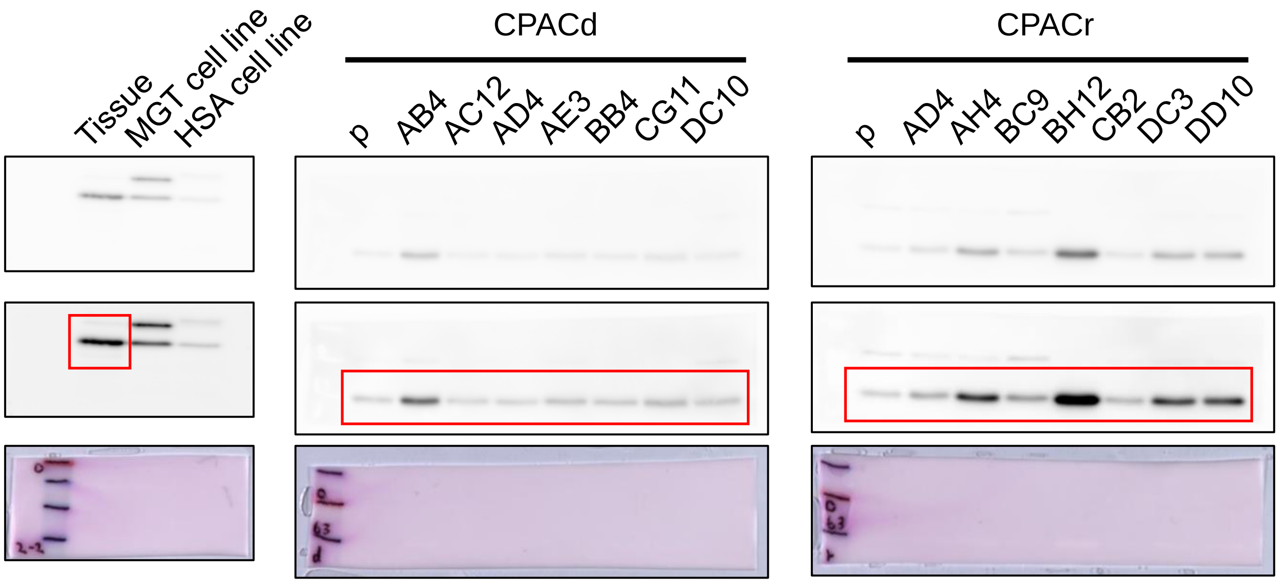

Supplementary Figures for Immunoblotting, continue

Immunoblotting images of Figure 5

#Total-Akt (60 kDa)

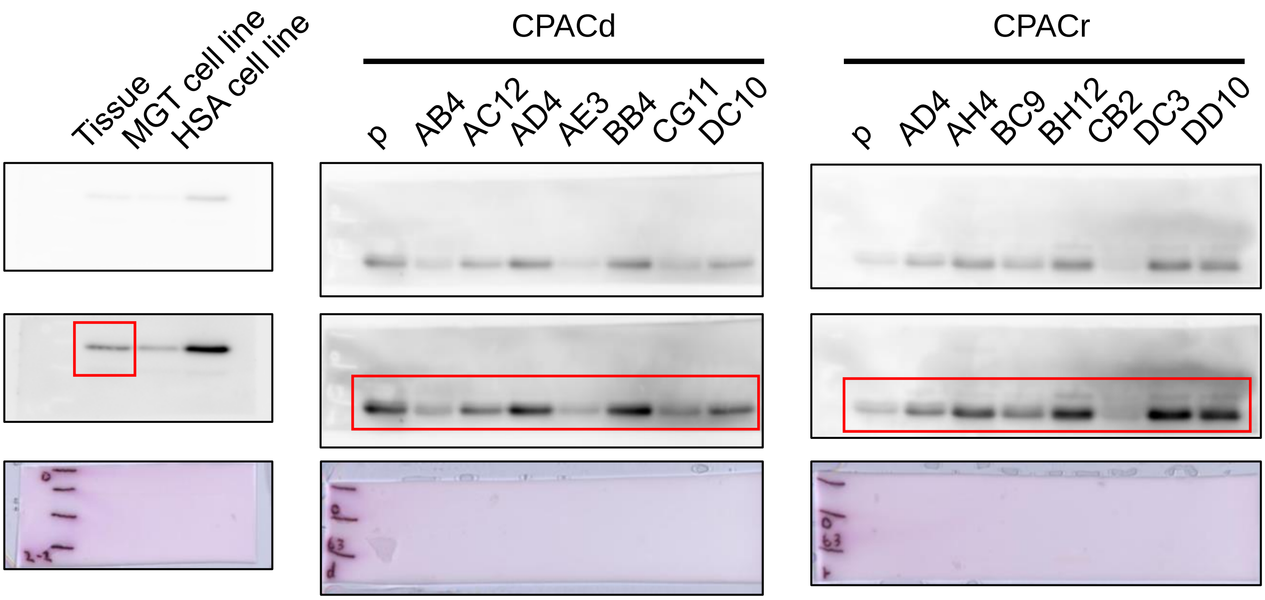

#β-actin (45kDa)

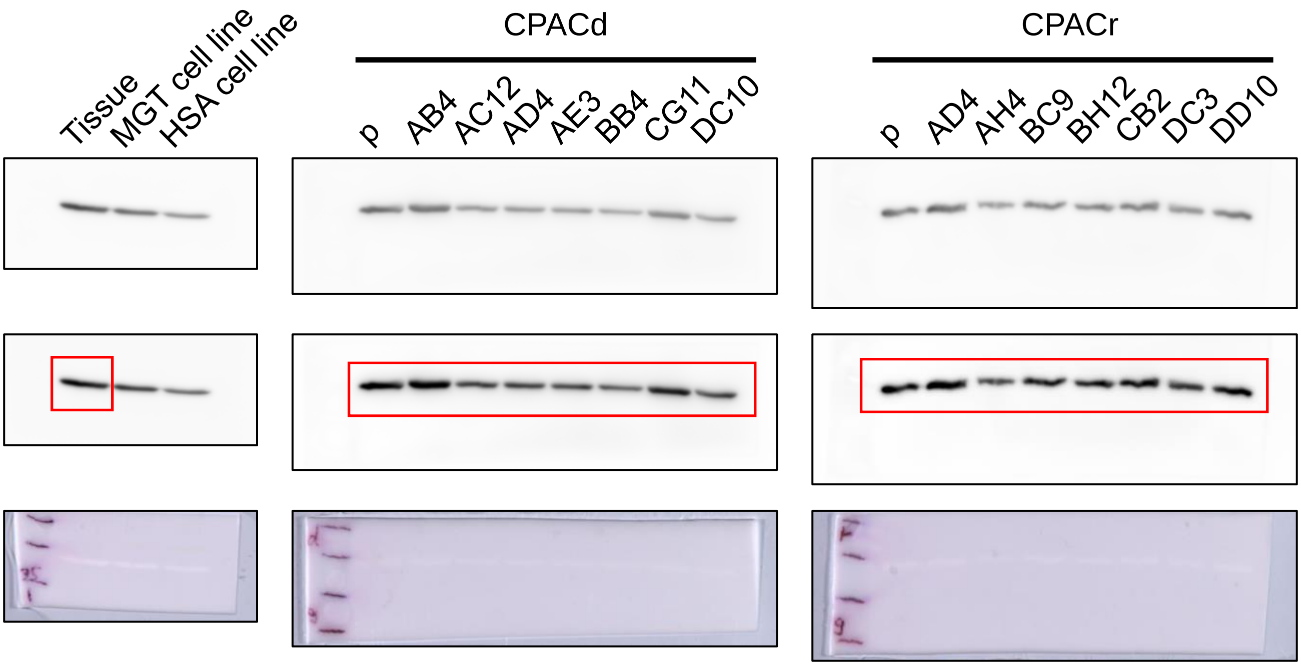

Supplement: Supplementary file 1 — Supplementary Information. [file 41598_2023_44062_MOESM1_ESM.pdf]
